# A Non-canonical Feedback Circuit for Rapid Interactions between Somatosensory Cortices

**DOI:** 10.1016/j.celrep.2018.04.115

**Published:** 2018-05-29

**Authors:** Genki Minamisawa, Sung Eun Kwon, Maxime Chevée, Solange P. Brown, Daniel H. O’Connor

**Affiliations:** 1 The Solomon H. Snyder Department of Neuroscience, Kavli Neuroscience Discovery Institute, The Johns Hopkins University School of Medicine, Baltimore, MD 21205, USA; 2 Brain Science Institute, The Johns Hopkins University School of Medicine, Baltimore, MD 21205, USA; 3 Biochemistry, Cellular and Molecular Biology Graduate Program, The Johns Hopkins University School of Medicine, Baltimore, MD 21205, USA

## Abstract

Sensory perception depends on interactions among cortical areas. These interactions are mediated by canonical patterns of connectivity in which higher areas send feedback projections to lower areas via neurons in superficial and deep layers. Here, we probed the circuit basis of interactions among two areas critical for touch perception in mice, whisker primary (wS1) and secondary (wS2) somatosensory cortices. Neurons in layer 4 of wS2 (S2_L4_) formed a major feedback pathway to wS1. Feedback from wS2 to wS1 was organized somatotopically. Spikes evoked by whisker deflections occurred nearly as rapidly in wS2 as in wS1, including among putative S2_L4_ → S1 feedback neurons. Axons from S2_L4_ → S1 neurons sent stimulus orientation-specific activity to wS1. Optogenetic excitation of S2_L4_ neurons modulated activity across both wS2 and wS1, while inhibition of S2_L4_ reduced orientation tuning among wS1 neurons. Thus, a non-canonical feedback circuit, originating in layer 4 of S2, rapidly modulates early tactile processing.

## In Brief

Minamisawa et al. uncover a rapid feedback pathway between secondary and primary somatosensory cortices that arises from layer 4 and acts to enhance sensory feature representation.

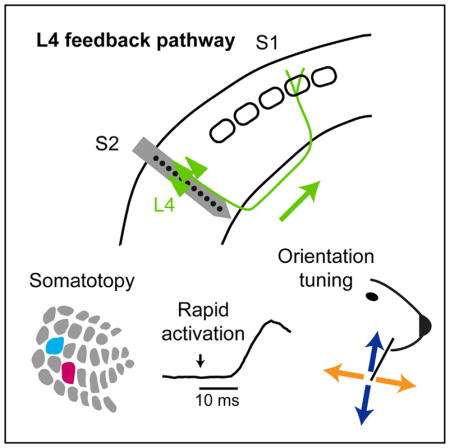

## INTRODUCTION

Sensory processing in cortex involves computations that occur both within and across cortical areas. The structure of long-range connections among these cortical areas exhibits motifs across mammalian species (Douglas and Martin, 2004; Harris and Shepherd, 2015). A canonical organization of feedforward and feedback connectivity has emerged from these recurring patterns of axonal projections. In the canonical circuit of sensory cortex, layer (L) 4 neurons receive inputs from earlier processing nodes, such as thalamus or lower (less integrative) cortical areas, and project mainly locally within a cortical area. Higher (more integrative) cortical areas, in addition to receiving input from lower areas, then send feedback projections to lower areas via output neurons located in superficial and deep cortical layers (Felleman and Van Essen, 1991; Markov and Kennedy, 2013; Rockland and Pandya, 1979). In the canonical circuit, L4 neurons act locally and do not originate feedback projections. Feedback projections are thought to mediate contextual influences on perception and be essential for multiple aspects of cortical computation and sensory coding.

Touch perception depends on dynamic interactions of primary (S1) and secondary (S2) somatosensory cortices. S2 in rodents, as in other species, is commonly agreed to be a higher brain area compared with S1, with receptive fields that are larger and more dependent on behavioral state (Brett-Green et al., 2004; Carvell and Simons, 1986; Chen et al., 2016; Krubitzer et al., 1986; Kwegyir-Afful and Keller, 2004; Kwon et al., 2016; Suter and Shepherd, 2015). Long-range axonal projections strongly interconnect the whisker representation regions of these cortical areas (wS1 and wS2, respectively) (Aronoff et al., 2010; Mao et al., 2011; White and DeAmicis, 1977). Both wS1 and wS2 are active during whisker-dependent behavior (Chen et al., 2016; Kwon et al., 2016; Zuo et al., 2015). However, much about the wS1 ↔ wS2 circuit and the computations it instantiates remains unknown.

Here, we report that a major feedback pathway from wS2 to wS1 originates in layer 4 of wS2. We find that projections between wS1 and wS2 are somatotopically aligned, such that areas of wS2 that receive input from a given part of the whisker map in wS1 project back to the same part of wS1. This reciprocal somatotopy suggests an anatomical substrate for dynamic feedforward and feedback interactions that preserve information about the stimulus. Stimulus-evoked activity occurred nearly as rapidly in wS2 as in wS1, including among putative S2_L4_ → S1 feedback neurons, suggesting that almost as soon as touch-evoked activity arrives in wS1 cortex, it is already subject to the influence of touch-evoked cortico-cortical feedback. Two-photon calcium imaging from S2_L4_ → S1 axons in superficial wS1 revealed that a subset of these axons send a signal back to wS1 that is selective for the orientation of whisker deflections. Optogenetic perturbations showed that S2_L4_ neurons modulate activity across both wS2 and wS1 and enhance orientation tuning in wS1. Our results uncover a non-canonical feedback circuit that exerts rapid and stimulus-specific regulation of cortical computations during touch.

## RESULTS

### A Major L4 Feedback Pathway within the S1 ↔S2 Circuit

**Figure 1:**
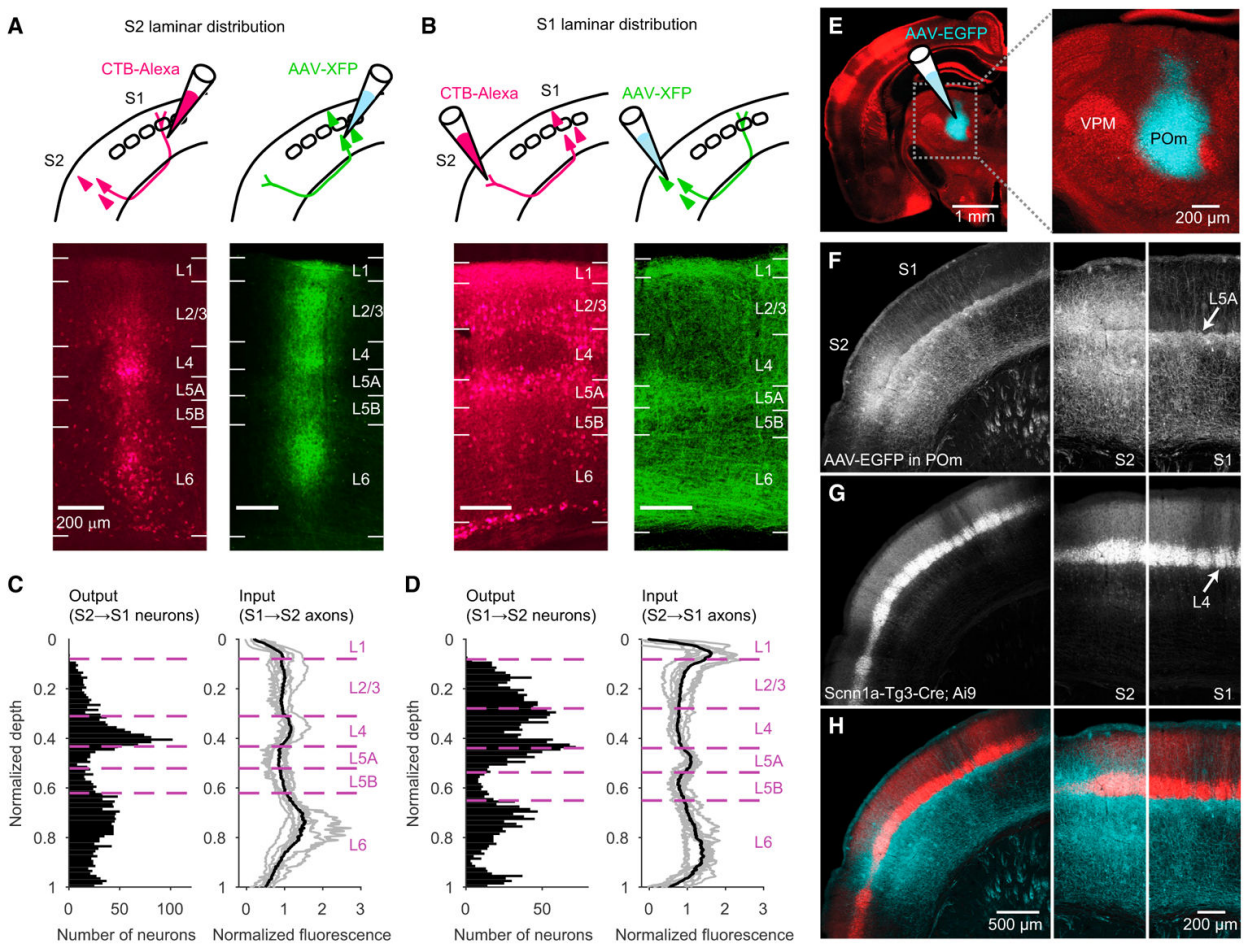
Layer 4 of S2 Provides a Major Output to S1 (A) Example images showing laminar distribution of labeling within wS2 after anterograde and retrograde tracer injections into wS1. Left: S2 → S1 neurons retrogradely labeled by CTB-Alexa injected into wS1. Layer boundaries were determined by GAD67 staining. Right: S1 → S2 axonal projections in wS2, anterogradely labeled by AAV tracer injected into wS1. (B) Similar to (A), but showing labeling within wS1 after anterograde and retrograde tracer injections into wS2. (C) Left: number of retrogradely labeled S2 →S1 neurons in wS2, plotted as a function of normalized depth within cortex (n = 2,830 neurons from 12 mice). Right: normalized S1 →S2 axonal fluorescence measured in wS2, shown for individual mice (gray curves, n = 8) and the mean (black). (D) Left: number of retrogradely labeled S1 →S2 neurons in wS1 (n = 2,624 neurons from 9 mice). L4 labeling reflects neurons located in septa (Figure S2). Right: normalized S2 →S1 axonal fluorescence measured in wS1 (n = 8 mice). (E) Coronal section from an Scnn1a-Tg3-Cre;Ai9 mouse injected in the thalamic posterior medial nucleus (POm) with AAV-EGFP. Right: zoom showing EGFP (cyan) in POm. The ventral posterior medial nucleus (VPM) is also indicated. (F) Thalamocortical axons from POm shown (EGFP fluorescence) in a coronal section spanning wS1 and wS2. A band of axons is visible in L5A of wS1 (arrow at far right), and can be seen to continue into wS2 (left and middle panels). In wS2, a second band of POm axons occurs superficial to the L5A band. Same animal as in (E). (G) tdTomato fluorescence from section in (F), showing L4 labeling in wS1 (arrow at far right), and the continuation of this band into wS2 (left and middle panels). (H) Overlay of EGFP and tdTomato fluorescence. Labeled neurons (red) in wS2 of the Scnn1a-Tg3-Cre;Ai9 mouse are located above L5A, in the band of POm axons corresponding to L4. See also Figures S1–S4.

We mapped the laminar pattern of corticocortical connectivity between wS2 and wS1 using targeted injections of anterograde and retrograde tracers. First, we localized wS2 and wS1 using intrinsic signal imaging. We then injected the retrograde tracer cholera toxin subunit B fused to fluorescent Alexa dyes (CTB-Alexa) into wS1 to label the cell bodies of neurons in wS2 that project to wS1 (S2 → S1 neurons; Figure 1A). In separate mice, we injected anterograde adeno-associated virus (AAV)-based fluorescent tracers (AAV-XFP) into wS1 to label axons projecting from wS1 to wS2 (S1 →S2 axons; Figure 1A). Similarly, we injected CTB-Alexa into wS2 to label S1 →S2 neurons, and AAV-XFP into wS2 to label S2 → S1 axons (Figure 1B). In coronal brain sections from wS1 and wS2, we counted retrogradely labeled cell bodies and quantified fluorescence from anterogradely labeled axons (Figure 1C and 1D). For each coronal section, we normalized cortical depth of all cell counts and fluorescence measurements based on the distance from pia to white matter and assigned laminar boundaries (STAR Methods).

Layer 4 was evident in both wS1 and wS2 based on genetic and immunohistochemical markers (Figure S1) (Hooks et al., 2011; Madisen et al., 2010; Meyer et al., 2010; Pouchelon et al., 2014; Yamawaki et al., 2014). The location of L4 in wS2 was further evident from the pattern of thalamocortical axons arising from the posterior medial nucleus (POm) (Figures 1E–1H). Specifically, injections of AAV-XFP into POm (Figure 1E) yielded axonal labeling in L4 of wS2 (Pouchelon et al., 2014; Viaene et al., 2011), as well as a distinguishable L5A band that extended across both wS1 (Kichula and Huntley, 2008; Lu and Lin, 1993; Wimmer et al., 2010), reflecting strong POm → L5A input (Bureau et al., 2006; Petreanu et al., 2009) and wS2 (Figures 1F and 1H). Neurons labeled in wS2 in the Scnn1a-Tg3-Cre mouse line (Madisen et al., 2010) (Figure 1G), which marks L4 neurons in wS1 (Madisen et al., 2010), fell within the L4 but not L5A band of POm axons (Figures 1H and S1E–S1L). Neurons labeled in the Scnn1a-Tg3-Cre line also overlapped with expression of the L4 marker RORβ (Liu et al., 2013) (Figure S1). Together, these observations indicate that the Scnn1a-Tg3-Cre line specifically labels L4 neurons in wS2.

Our results confirmed multiple features of the expected corticocortical connectivity pattern between wS1 and wS2. These included dense S2 →S1 axonal labeling in layer 1 of S1 (Cauller et al., 1998), typical of “top-down” inputs from higher to lower cortical areas (Figure 1B) (Larkum, 2013), and minimal retrograde labeling of L4 neurons in wS1 following CTB-Alexa injections in wS2 (Figures 1B, 1D, and S2), consistent with their projecting almost entirely within their home cortical column (Feldmeyer, 2012; Petersen, 2007). Remarkably, neurons labeled by CTB-Alexa injections in wS1 were abundant in L4 of wS2 (Figures 1A and 1C), indicating a major S2 → S1 feedback pathway originating from L4 of wS2 (we term this the S2_L4_ → S1 pathway). We confirmed this retrograde S2_L4_ → S1 labeling in separate experiments using a retrograde Cre-expressing AAV tracer (rAAV2-retro-Syn-Cre) (Tervo et al., 2016) injected into wS1 of Cre reporter mice (Ai9) (Madisen et al., 2010) (Figure S3). Moreover, injections of Cre-dependent AAV anterograde tracer (AAV-Flex-tdTomato) into wS2 of an L4 Cre mouse line (Scnn1a-Tg3-Cre) (Madisen et al., 2010) yielded prominent axonal fluorescence in wS1, particularly in layers 5A, 2/3, and 1 (Figures S4A–S4C).

Most neurons in wS1 L4 are spiny stellate neurons (Lefort et al., 2009). To examine the morphology of S2_L4_ → S1 neurons, we used injections of dilute AAV-Flex-tdTomato virus into wS2 of Scnn1a-Tg3-Cre mice, combined with injections of CTB-Alexa into wS1 of the same mice (Figure S4D). This resulted in both sparse labeling of S2_L4_ neurons with tdTomato and retrograde labeling of S2 → S1 neurons with CTB-Alexa (Figure S4D). Prominent apical dendrites could be identified based on confocal microscopy for a large fraction of both retrogradely labeled and unlabeled S2_L4_ neurons (Figures S4D and S4E), indicating that the S2_L4_ → S1 pathway originates at least in large part from L4 neurons with pyramidal morphology.

### Feedback from wS2 to wS1 Is Somatotopically Specific

**Figure 2:**
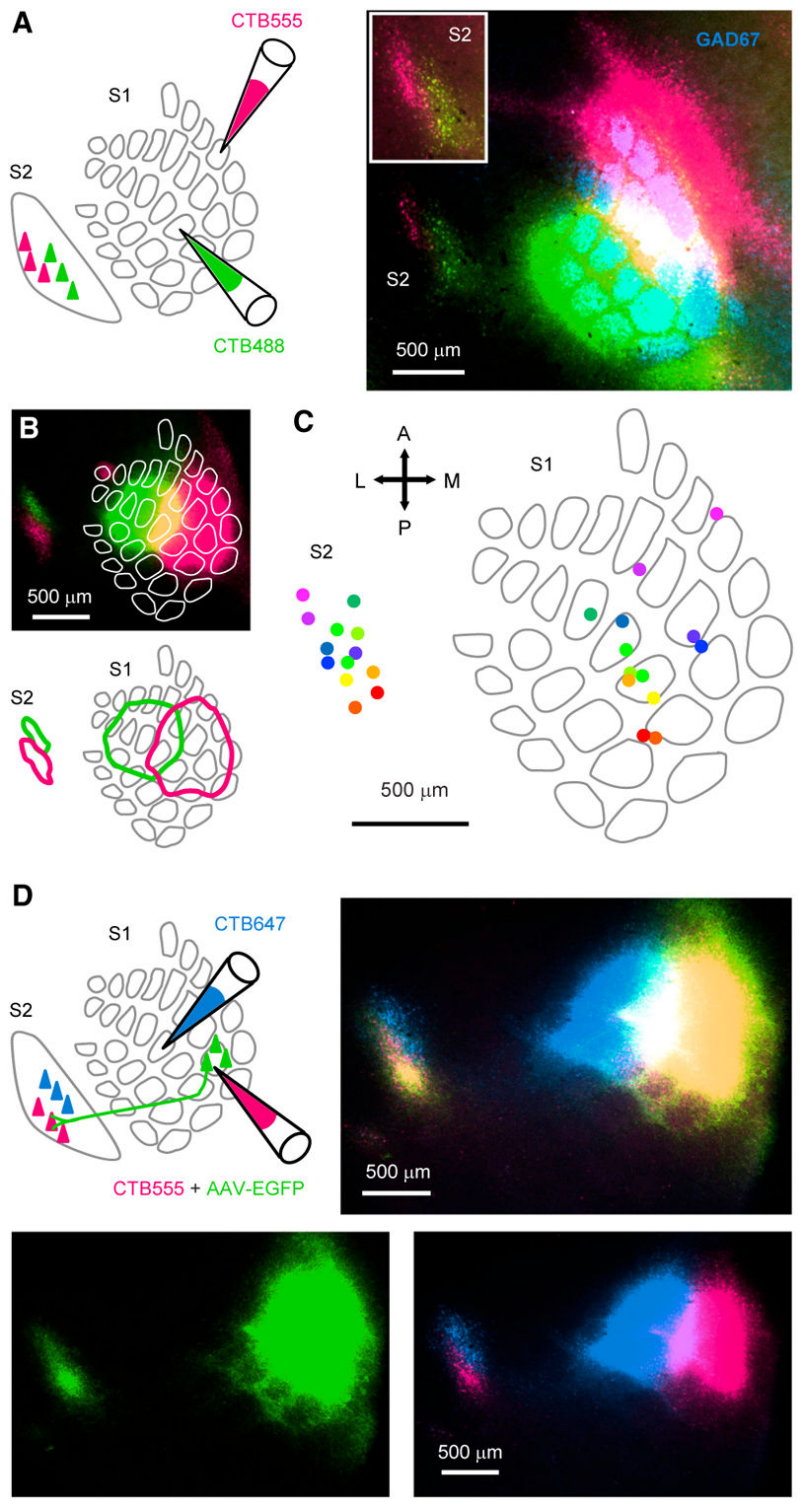
Feedback from S2 to S1 Is Somatotopic within the Whisker Regions (A) Two colors of CTB-Alexa were injected in different barrel columns of wS1, to retrogradely label neurons in wS2. The brain was sectioned tangentially and stained for GAD67 to show barrels in wS1. Inset: retrogradely labeled neurons in wS2 shown with higher image brightness. (B) Top: additional example of experiment in (A) from a different animal and with overlay of barrel map estimated from GAD67 staining. Bottom: outlines showing locations of CTB-Alexa injections in wS1 and extent of retrograde labeling in wS2. Outlines were used to determine the center of injection sites and retrogradely labeled sites across different animals. (C) Aggregate map across injections and mice (n = 13 injections in 7 mice) showing centers of injection sites in wS1 and retrogradely labeled locations in wS2 (corresponding sites in wS1 and wS2 indicated by colors). (D) Top left: a cocktail of retrograde (CTB-Alexa555) and anterograde (AAV-EGFP) tracers was injected at one site in wS1 and a different color of retrograde tracer (CTB-Alexa647) at another site in wS1. Top right: overlay of CTB and AAV-EGFP labeling in tangential sections shows reciprocal connections between wS1 and wS2. Bottom left: EGFP signal alone. Bottom right: CTB-Alexa signals. See also Figure S5.

Multiple forms of neural computation and top-down modulation are topographically specific. We sought to determine whether the S2 ↔S1 circuit provides a basis for topographically specific interactions within the regions that represent the whiskers. Feed-forward connectivity from wS1 to wS2 is topographically specific, in that nearby regions of wS1 project to nearby regions of wS2 (Hoffer et al., 2003; Mao et al., 2011). Moreover, co-injection of retrograde and anterograde tracers into a single site in wS1 results in co-labeling in wS2 (Aronoff et al., 2010). These and other prior studies that compared patterns of retrograde S2 → S1 labeling across animals (Carvell and Simons, 1987) or across large portions of the body maps (Fabri and Burton, 1991) imply, but do not directly demonstrate, that wS2 → wS1 feedback is topographically specific. A direct demonstration requires evidence that nearby regions of wS2 project to nearby regions of wS1. To determine whether wS2 →wS1 feedback is topographically specific, we injected two colors of the retrograde tracer CTB-Alexa into nearby regions of wS1 (Figure 2A; injections were targeted two barrel columns apart, e.g., C1 and C3 columns). In flattened histological sections we observed retrogradely labeled cell bodies within wS2, with tracer from the two injection sites located in adjacent patches of wS2 (Figures 2A and 2B). For each injection, we determined the approximate center of the fluorescent labeling at the injection site in wS1 and of the retrogradely labeled patch in wS2 (Figures 2B and 2C). Across mice, a somatotopic pattern of retrograde labeling in wS2 was apparent, in which pairs of injection and labeled sites were “reflected” about an axis separating wS2 from wS1 (Figure 2C). Specifically, posterior injection sites in wS1 resulted in posterior labeling in wS2, while anterior injection sites in wS1 resulted in anterior labeling in wS2. Medial injection sites in wS1-labeled lateral wS2, while lateral injection sites in wS1-labeled medial sites in wS2. This pattern of retrograde labeling matches the somatotopic organization of rodent S2 (Carvell and Simons, 1986, 1987; Kleinfeld and Delaney, 1996; Koralek et al., 1990; Krubitzer et al., 1986; Remple et al., 2003). Thus, our data suggest that at least partially distinct locations in wS2 project to wS1 sites that are somatotopically matched (represent the same whiskers). In separate experiments, we sectioned brains coronally and found that this somatotopic pattern of retrograde labeling included L4 (Figures S5A–S5D; n = 2 mice). Finally, in additional experiments, we injected one site within wS1 with CTB-Alexa647 and a nearby wS1 site with CTB-Alexa555 and the anterograde tracer AAV-EGFP (Figure 2D). Fluorescence from the co-injected retrograde and anterograde tracers overlapped within wS2 (Figures 2D, S5E, and S5F; n = 3 mice), indicating that regions of wS2 receiving input from a region of wS1 send projections back to the same region of wS1. Thus, the whisker regions of S1 and S2 are interconnected in a somatotopically specific loop.

### Rapid and Orientation-Specific Responses to Touch among S2_L4_ → S1 Neurons

**Figure 3:**
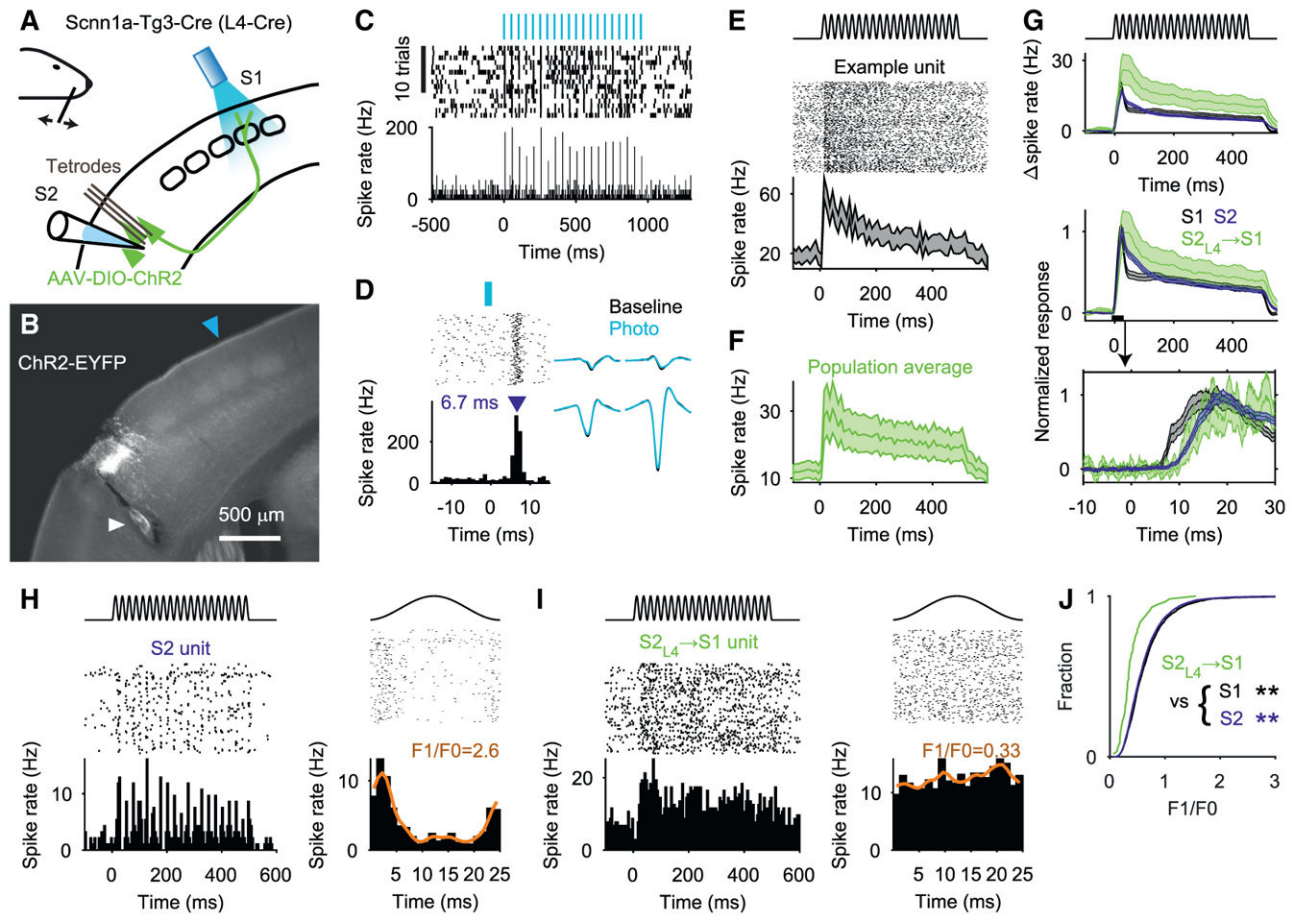
Putative S2_L4_→S1 Neurons Are Rapidly Excited by Touch (A) Tetrodes were used to record wS2 single-unit responses to whisker deflections in Scnn1a-Tg3-Cre mice injected with AAV-DIO-hChR2-EYFP into wS2. At the end of each recording session, trains of blue light pulses (2 ms pulses at 20 Hz) were applied over wS1 in order to antidromically stimulate putative S2_L4_ → S1 neurons. (B) Expression of ChR2-EYFP in S2_L4_. Blue arrowhead: estimated location of the optic fiber tip over wS1. White arrowhead: electrolytic lesion used to locate the tetrode tract. (C) Example spike raster and spike time histogram showing activity evoked by light pulses (indicated at top) for a putative S2_L4_ → S1 unit. (D) Left: raster and PSTH for unit in (C) showing responses to all laser pulses within a shorter peri-stimulus time window. Blue arrowhead: spike latency (6.7 ms) measured from light onset to PSTH peak. Right: spike waveform means from the four tetrode channels, for spikes occurring in the absence (“Baseline”) of or in response to (“Photo”) light pulses. (E) Top: schematic of the sinusoidal whisker deflection waveform (40 Hz, 0.5 s). Middle: spike raster for a putative S2_L4_ → S1 unit (first 100 trials). Bottom: PSTH (95% CI) for the example unit. (F) Population average PSTH from putative S2_L4_ → S1 units (±95% CI; n = 58 units from 6 mice). (G) Top: overlay of population average PSTHs (±95% CIs) for those wS1 (gray; n = 585), wS2 (blue; n = 1,557), and putative S2_L4_ →S1 (green; n = 39) units excited within the first 50 ms after stimulus onset. Middle: plot at top is shown after normalizing each PSTH to its peak. Bottom: zoom of time window near stimulus onset. (H) Left: spike raster (first 100 trials) and PSTH for an example wS2 unit with reliable response to 40 Hz whisker deflection waveform. Right: stimulus cycle-locked raster plot and PSTH for same unit. Orange curve: kernel density estimate used to quantify the range (F1) and mean (F0) of the average response to one cycle of the whisker stimulus. The F1/F0 ratio quantifies the degree of cycle-by-cycle spike rate modulation for each unit. (I) Same as (H) but for a putative S2_L4_ → S1 unit without clear cycle-by-cycle modulation. (J) Cumulative histograms showing the F1/F0 ratio for all wS1 (n = 1,841), wS2 (n = 5,557), and putative S2_L4_ → S1 (n = 58) units. S2_L4_ → S1 units showed lower values compared with wS1 (**p = 4.4 × 10^−9^; Wilcoxon rank-sum) and wS2 (**p = 1.4 × 10^−7^; Wilcoxon rank-sum) units.

To address whether wS2 neurons that project back to wS1 respond to whisker touch, and could thus potentially serve as a somatotopically specific feedback signal, we focused on the S2_L4_ → S1 pathway. We used injections of virus (AAV-DIO-hChR2-EFGP) in mice expressing Cre recombinase in L4 neurons (Scnn1a-Tg3-Cre) to introduce channelrhodopsin-2 (ChR2) into S2_L4_ neurons. We implanted 32-channel tetrode arrays into wS2 and an optic fiber over wS1 (Figures 3A and 3B). In awake, head-fixed mice, we delivered pulses of blue light over wS1 to photostimulate S2_L4_ → S1 neurons via excitation of their axons in wS1. We found neurons in wS2 that responded at short latency (6.5 ± 2.2 ms, n = 58) following pulses of light delivered to wS1 (pulse duration: 2 ms, in trains of 20 pulses at 20 Hz; Figures 3C and 3D). Action potentials occurring spontaneously and in response to light had matching waveforms (Figure 3D). These single units we refer to as “putative” S2_L4_ → S1 neurons (n = 58 units from 6 mice).

Putative S2_L4_ → S1 neurons were excited robustly by our whisker stimulus (40 Hz, 0.5 s sinusoidal deflections) (Figures 3E and 3F). To compare the responses of S2_L4_ → S1 neurons with large populations of wS2 and wS1 neurons, we first pooled data separately for wS2 and wS1 units obtained from: (1) the general population of wS2 units from tetrode recordings, including the subset of recordings in which we obtained putative S2_L4_ → S1 neurons (4,501 units total from 21 mice; presumably including unlabeled S2_L4_ → S1 neurons); (2) tetrode recordings obtained from wS1 (873 units total from 10 mice, including 7 of the mice used for wS2 recordings, in which wS1 was recorded simultaneously with wS2); and (3) recordings from 64-channel laminar silicon probe arrays implanted into wS2 (n = 1,056 units from 12 mice) or wS1 (n = 968 from 13 mice; silicon probe data taken from experiments reported in Figures 5, 6, and 7).

Could activity among S2_L4_ → S1 neurons impact the early phases of tactile stimulus processing in wS1 and wS2? To address this question, we compared the stimulus response latency for the population of putative S2_L4_ → S1 neurons with those of the larger populations of rapidly excited wS1 and wS2 neurons, defined as those neurons excited by the whisker stimulus within the first 50 ms after stimulus onset (39 of 58 putative S2_L4_ → S1 units; 1,557 of 5,557 wS2 units; 585 of 1,841 wS1 units). The putative S2_L4_ →S1 neurons responded within the first 10 ms following onset of the whisker stimulus (95% confidence interval [CI] exceeded baseline spike rate at 10.3 ms), similar to the general wS2 (7.6 ms) and wS1 (6.1 ms) populations (Figure 3G). Thus, spike rates among wS1, wS2, and S2_L4_ →S1 neurons all increase rapidly following stimulus onset (see also Kwegyir-Afful and Keller, 2004; Liao and Yen, 2008; Melzer et al., 2006). This short latency excitation of wS2 could arise in part from direct inputs from ventral basal thalamus to wS2 (Carvell and Simons, 1987; Feldmeyer, 2012; Pierret et al., 2000) or from strong, monosynaptic S1 → S2 feedforward excitation (wS1 activity occurred slightly earlier, with 95% CIs for wS1 and wS2 no longer overlapping by 6.4 ms) (Figure 3G). The rapid onset of activity across the wS1, wS2, and putative S2_L4_ → S1 populations suggests that wS1 and wS2 participate in nearly simultaneous aspects of computation related to incoming sensory information, with interactions mediated in part by the S2_L4_ → S1 pathway.

Compared with the general wS2 (n = 5,557) and wS1 (n = 1,841) populations, however, putative S2_L4_ → S1 neurons (n = 58) showed spike rates less modulated by individual stimulus cycles (Figures 3H–3J).

**Figure 4:**
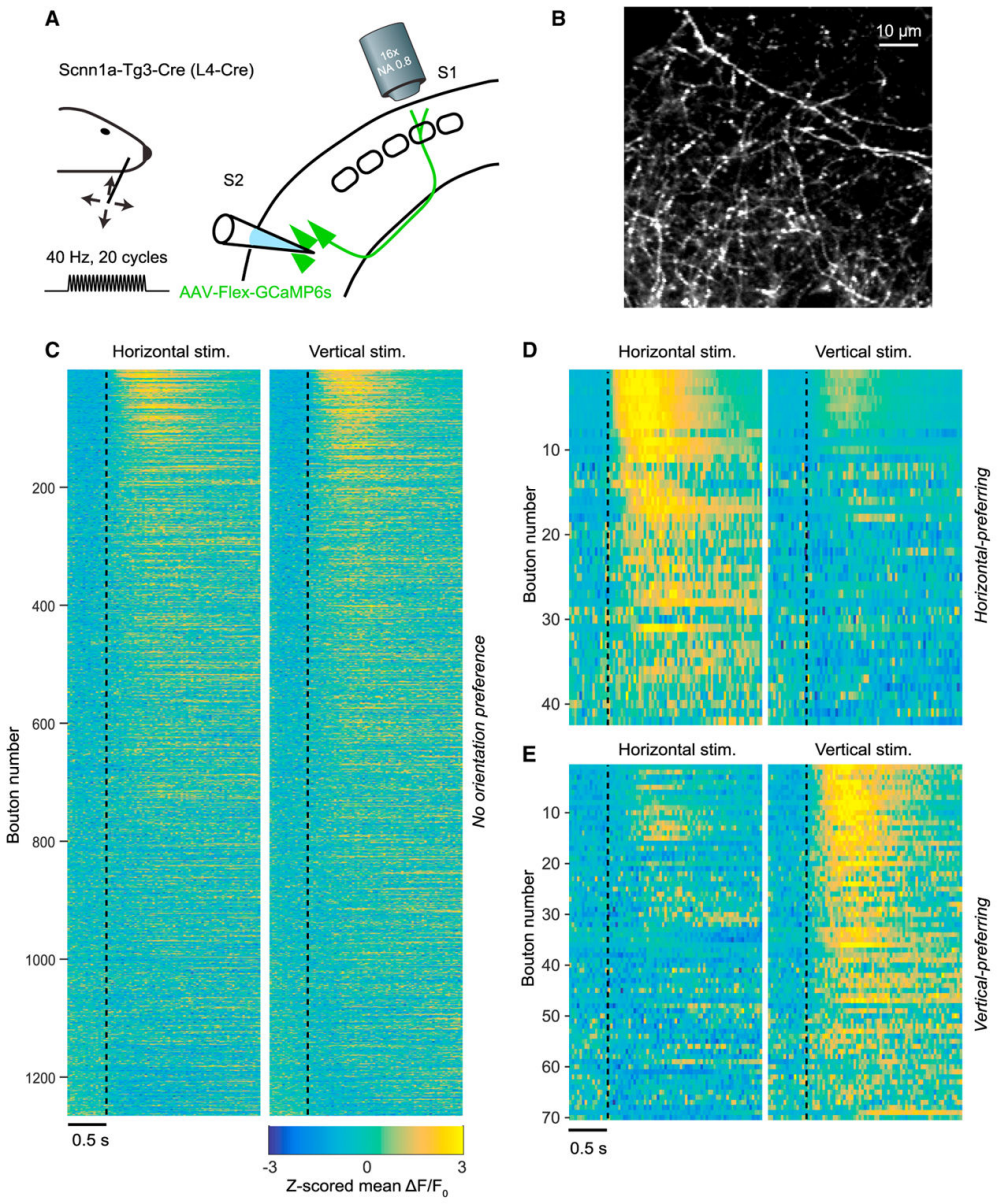
S2_L4_ → S1 Neurons Send Orientation-Specific Activity to S1 (A) Two-photon calcium imaging of S2_L4_ → S1 axons in superficial S1 was performed during sinusoidal deflections (40 Hz, 0.5 s) of a single whisker along either horizontal or vertical orientations. (B) Example field-of-view (FOV) showing GCaMP6s-labeled S2_L4_ → S1 axons in S1 (mean fluorescence across one trial). (C) Heatmaps of *Z* scored responses of each axonal bouton (along rows) to either horizontal (left) or vertical (right) whisker deflections, for boutons that showed similar responses to the two orientations (n = 1,265 boutons from 12 FOVs in 4 mice). Dashed vertical lines: onset of whisker stimulus. (D) Same as (C) but for boutons with significantly larger responses for horizontal compared with vertical deflections (n = 42 from 11 FOVs in 4 mice). (E) Same as (C) but for boutons with significantly larger responses for vertical deflections (n = 70 from 12 FOVs in 4 mice).

To confirm directly that S2_L4_ neurons propagated activity to wS1, we performed two-photon imaging of axons from S2_L4_ neurons within superficial layers of S1 (Figure 4) in awake mice. We injected AAV expressing the calcium indicator GCaMP6s (Chen et al., 2013) in a Cre-dependent manner in wS2 of Scnn1a-Tg3-Cre mice. In subsequent imaging sessions, we delivered whisker deflections along orthogonal orientations corresponding to motion in approximately the horizontal (azimuthal) or vertical (elevational) planes while imaging axonal boutons in wS1 (Figures 4A and 4B). Whisker stimulation evoked changes in fluorescence among S2_L4_ → S1 boutons (Figure 4C). A subset of boutons showed orientation-specific responses, with larger fluorescence changes for either horizontal (Figure 4D) or vertical (Figure 4E) whisker deflections. Thus, S2_L4_ sends a rapidly evoked and orientation-tuned sensory response to wS1.

### S2_L4_ Neurons Modulate Activity in Both wS2 and wS1

**Figure 5:**
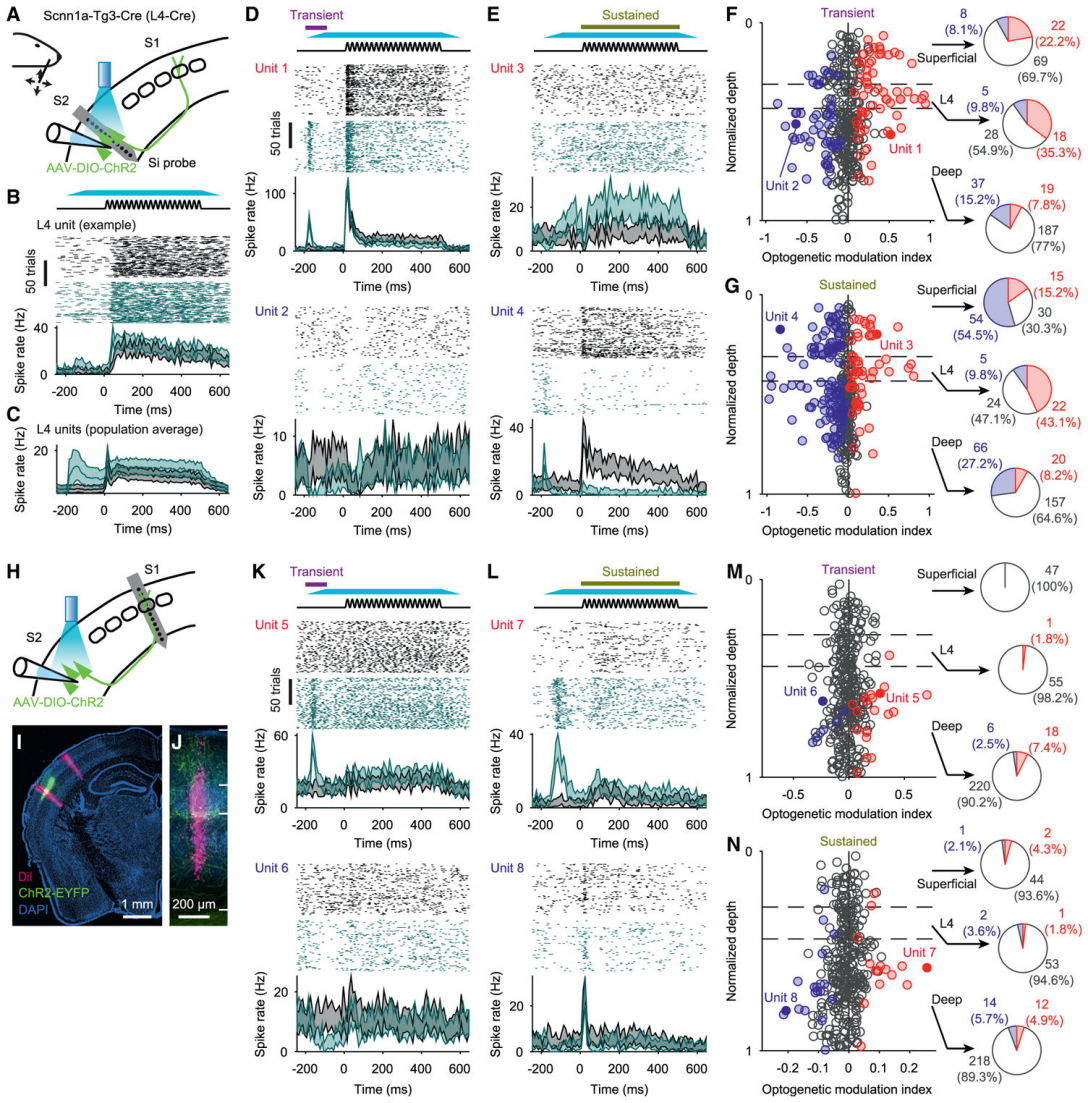
Excitation of S2_L4_ Neurons Modulates Activity across Layers in S2 and S1 (A) Schematic of wS2 recording with S2_L4_ stimulation. Silicon probes recorded single units across layers in wS2. Responses were recorded to sinusoidal deflections of a single whisker along either horizontal or vertical orientations, and to optogenetic excitation (via ChR2) of S2_L4_ neurons that began prior to and spanned the duration of the whisker stimulus. (B) Top: schematic of the whisker stimulus and 470 nm illumination (dark cyan). Middle: spike rasters for an S2_L4_ unit with (green) and without (black) optogenetic stimulation of S2_L4_ (first 100 trials for each). Bottom: PSTHs (95% CI) for the example unit with (green) and without (black) optogenetic stimulation. (C) Population average PSTHs from S2_L4_ units with (green) and without (black) optogenetic stimulation (±95% CI; n = 51 units from 6 mice). (D) Example units with significantly higher (unit 1, at top) or lower (unit 2, at bottom) spike rates during a “transient” phase of the optogenetic stimulus (indicated by purple horizontal bar at top; first 100 ms of LED illumination, from −200 ms to −100 ms relative to whisker stimulus onset). Conventions as in (B). (E) Example units with significantly higher (unit 3, at top) or lower (unit 4, at bottom) spike rates during a “sustained” phase of the optogenetic stimulus (indicated by brown horizontal bar at top; 500 ms covering identical period as the whisker stimulus). Conventions as in (B). (F) Optogenetic modulation index for the transient phase of the optogenetic stimulus, plotted for each unit as a function of normalized depth within cortex. Estimated boundaries of cortical layer 4 are indicated with horizontal dashed lines. Filled circles indicate significantly excited (red) or inhibited (blue) units. Example units from (D) are indicated (dark red and dark blue filled circles). Pie charts show percentages of units with significant optogenetic modulation for L4 and layers above (“superficial”) and below (“deep”) L4 (n = 393 units from 6 mice). (G) Same as (F) but for the sustained phase of optogenetic stimulation. (H) Schematic of wS1 recording with S2_L4_ stimulation. Experiments were identical to those of (A)–(G) except recordings were made in wS1. (I) Coronal section showing DiI-marked silicon probe recording tracts (magenta) in wS2 (left tract) and wS1 (right tract), and ChR2-EYFP fluorescence in S2_L4_. (J) Zoom of (I) showing wS2 tract and estimated pia, L4, and white matter boundaries (white horizontal lines). (K–N) Same as (D)–(G) but for wS1 instead of wS2 recordings (n = 347 units from 6 mice). See also Figure S6.

What impact does S2_L4_ activity have locally in wS2 and in wS1? To address this question, we recorded single units in wS2 or wS1 using silicon probe arrays and optogenetically stimulated S2_L4_ neurons in awake, head-fixed mice (Figure 5). We injected AAV expressing ChR2 in a Cre-dependent manner into wS2 of Scnn1a-Tg3-Cre mice and positioned an optic fiber over wS2. At the time of electrophysiology experiments, an acute 64-channel silicon probe was inserted into either wS2 (Figure 5A) or into wS1 (Figure 5H). Responses to whisker deflections were obtained from single units under baseline conditions and during periods of ChR2 photostimulation (ramped onset and offset with constant illumination spanning from 100 ms before stimulus onset to 500 ms afterward). We assigned units to either “superficial,” “L4,” or “deep” categories based on cortical depth and current source density analysis. In wS2, units categorized as L4 were excited by the photostimulus, as expected (Figure 5B). Across the population of S2_L4_ units, we observed transient responses to the ramped onset of the photostimulus (during which there was no whisker deflection), as well as sustained responses to the steady-state portion of the photostimulus (quantified from 0–500 ms following onset of the whisker stimulus; Figure 5C). In superficial and deep layers of wS2, units also responded to the transient (Figures 5D and 5F) and sustained (Figures 5E and 5G) portions of the photostimulus (transient and sustained responses were correlated) (Figure S6). Although these superficial and deep wS2 units responded in a heterogeneous manner whereby individual units could be either excited or inhibited by L4 excitation (Figures 5D–5G), ~3.5 times as many units were inhibited by the sustained stimulus as were excited. Overall, 212 of 393 units total in wS2 (n = 6 mice) were significantly modulated by the photostimulus (either the transient or sustained portions) (Figure S6). In wS1, a much smaller fraction of units showed significant responses to S2_L4_ photostimulation (50 of 347 units total from 6 mice) (Figures 5H–5N and S6). Among those wS1 units that were significantly modulated, most were in deep layers (Figures 5M and 5N). These wS1 units showed both transient and sustained responses to S2_L4_ photostimulation and included both increases and decreases in activity (Figures 5K–5N).

**Figure 6:**
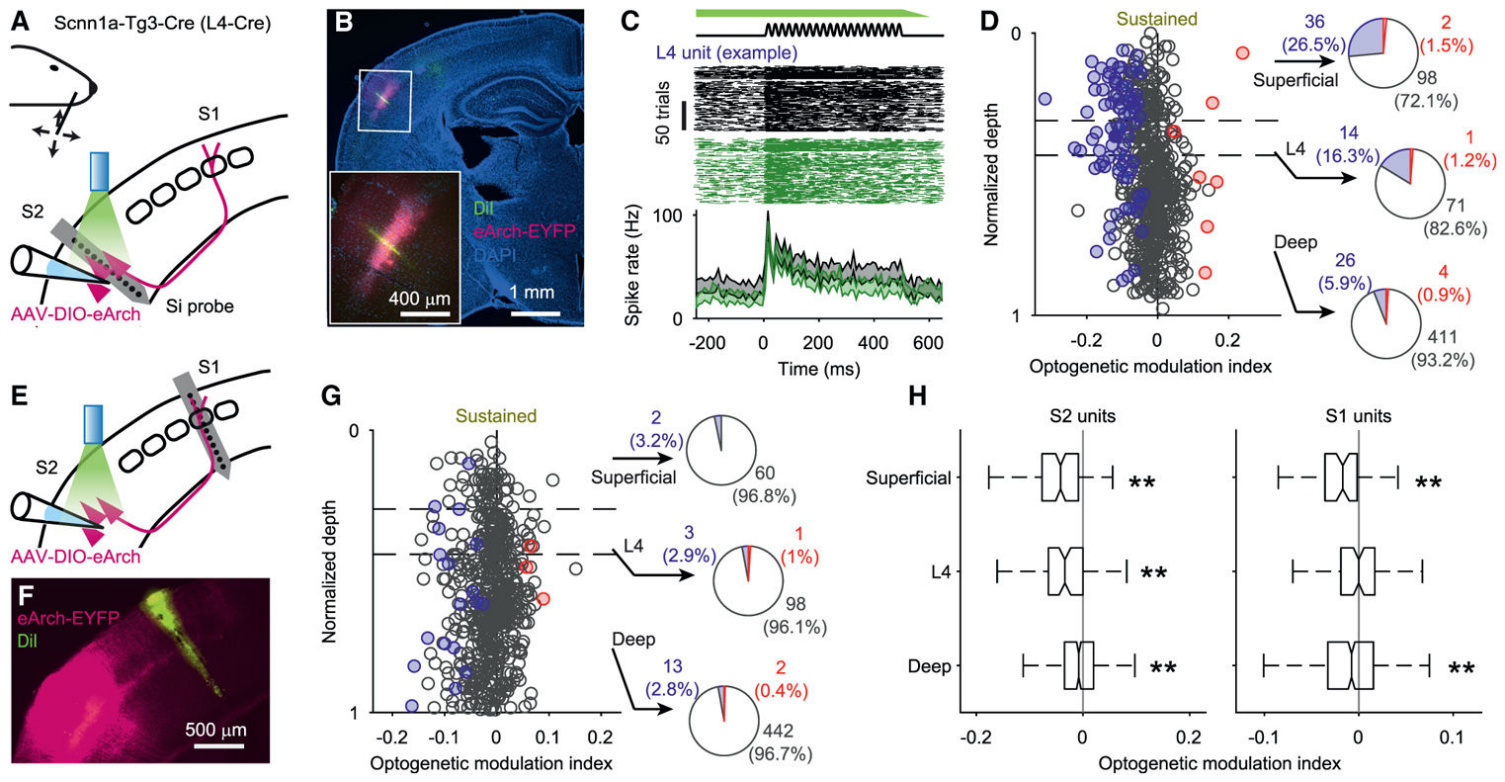
Inhibition of S2_L4_ Neurons Suppresses Activity in S2 but Only Weakly So in S1 (A) Schematic of wS2 recording with S2_L4_ inhibition. Silicon probes recorded single units across layers in wS2. Responses were recorded to sinusoidal deflections of a single whisker along either horizontal or vertical orientations, with and without optogenetic inhibition (via eArch3.0) of S2_L4_ that began prior to and spanned the duration of the whisker stimulus. (B) Coronal section showing DiI-marked silicon probe tract (green) in wS2, and eArch3.0-EYFP fluorescence in S2_L4_. Inset: zoom of region in white box. (C) Top: schematic of the whisker stimulus and 565 nm illumination (light green; preceding ramp-up not depicted). Middle: spike rasters for an S2_L4_ unit with (green) and without (black) optogenetic inhibition of S2_L4_ (first 100 trials for each). Bottom: PSTHs (95% CI) for the example unit with (green) and without (black) inhibition. (D) Optogenetic modulation index for the sustained phase (same time period as the whisker stimulus) of the optogenetic inhibition, plotted for each unit as a function of normalized depth within wS2 (n = 663 units from 6 mice). Conventions as in Figure 5F. Example L4 unit from (C) indicated by dark blue filled circle. (E) Schematic of wS1 recording with S2_L4_ inhibition. Experiments were identical to those depicted in (A)–(D) except silicon probe recordings were made in wS1. (F) Coronal section showing DiI-marked silicon probe recording tract (green) in wS1 and eArch3.0-EYFP fluorescence in S2_L4_. (G) Same as (D) but for wS1 recordings (n = 621 units from 7 mice). (H) Boxplots for wS2 (left) and wS1 (right) units depicting 25th, 50th, 75th percentiles and range of the optogenetic modulation index after removal of outliers (STAR Methods; **, wS2: superficial, p = 2.3 × 10^−14^; L4: p = 5.0 × 10^−8^, deep: p = 1.4 × 10^−3^; wS1: superficial: p = 5.8 × 10^−5^, L4: p = 1, deep: p = 3.7 × 10^−4^; sign tests).

In complimentary experiments, we inhibited S2_L4_ neurons using eArch3.0 (Chow et al., 2010; Mattis et al., 2011) following AAV injections into wS2 of Scnn1a-Tg3-Cre mice, while recording single-unit responses to whisker deflections in either wS2 or wS1 (Figure 6). Horizontal and vertical deflections were pooled for initial analyses (those shown in Figure 6). Illumination of wS2 (565 nm) led to a partial inhibition of S2_L4_ units (Figures 6C and 6D). Approximately 16% (14 of 86) of S2_L4_ units showed an inhibition of the whisker stimulus response that was statistically significant at the single neuron level (Figure 6D). The distribution of an optogenetic modulation index across the population of units was shifted to negative values, indicating that many units that did not individually reach statistical significance were nonetheless inhibited (Figure 6H, left). One quarter of superficial units (~26%, 36 of 136) in wS2 were also inhibited by S2_L4_ inhibition, as was a smaller fraction (~6%, 26 of 441) of deep units. Thus, S2_L4_ neurons act to increase activity during whisker stimulation throughout the local wS2 cortical column. In wS1, only a very small fraction of individual units were significantly modulated (21 of 621 total; Figure 6G). However, at the population level, the optogenetic modulation index was significantly shifted to negative (inhibited) values for superficial and deep layers but not L4 (Figure 6H, right). These results indicate that the effect of modest S2_L4_ inhibition on wS1 of awake (but not task performing) mice is a widespread but weak reduction in overall activity across superficial and deep layers.

### S2_L4_ Neurons Enhance Orientation Sensitivity in wS1

**Figure 7:**
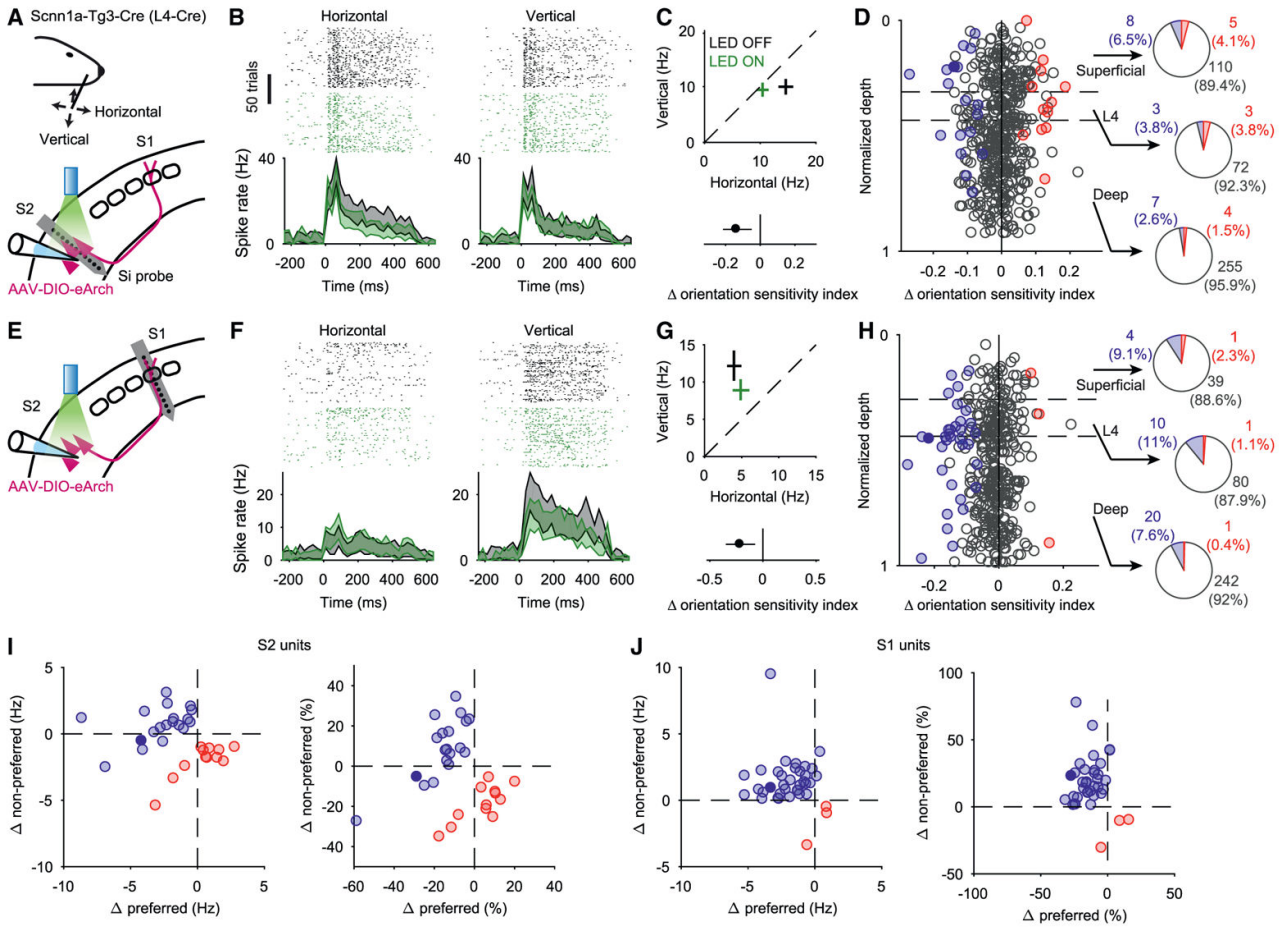
Inhibition of S2_L4_ Neurons Reduces Orientation Sensitivity in S1 (A) Schematic of experiment: same as in Figure 6A, but with analysis (B)–(D) limited to whisker-responsive units. (B) Spike rasters (first 100 trials) and PSTHs (95% CIs) showing responses to horizontal (left column) or vertical (right) whisker stimulations for a wS2 unit with (green) and without (black) inhibition of S2_L4_. (C) Top: spike rate during horizontal versus vertical whisker deflections (means ± 95% CIs) for the example unit in (B). Bottom: change in orientation sensitivity index (*OSI*; ±95% CI) due to optogenetic inhibition for same unit. (D) Change in *OSI* plotted for each whisker-responsive wS2 unit as a function of normalized depth within cortex. Conventions as in Figure 5F. Example unit from (B) indicated by dark blue filled circle. Pie charts show percentages of wS2 units with significant change in *OSI* during S2_L4_ optogenetic inhibition (n = 467 whisker-responsive units from 6 mice). (E) Schematic of experiment: same as in Figure 6E, but with analysis (F)–(H) limited to whisker-responsive units. (F and G) Same as (B) and (C) but for a wS1 unit. (H) Same as (D) but for wS1 units (n = 398 whisker-responsive units from 7 mice). Example unit from (F) indicated by dark blue filled circle. (I) Left: change in mean spike rate during inhibition of S2_L4_ for preferred and non-preferred whisker deflection orientations, for those wS2 units (filled circles from D) showing significantly increased (red; n = 12) or decreased (blue; n = 18) *OSI*. Right: same data as in left panel but expressed as percent change in firing rate. (J) Same as (I) but for wS1 units (n = 3 and 34 units for increased and decreased *OSI*, respectively). See also Figure S7.

The orientation or direction of whisker deflections is thought to be a stimulus feature of major significance for rodent somato-sensation, critical to interpret the complex interactions (Hobbs et al., 2016) that occur between whiskers and objects. To test whether the S2_L4_ → S1 pathway plays a role in shaping orientation tuning, we analyzed our data to examine the effect of S2_L4_ inhibition on orientation-specific responses in wS2 and in wS1 (Figure 7). An orientation sensitivity index (*OSI*) quantified the degree to which each unit preferred either horizontal or vertical whisker deflections. Under baseline conditions, large fractions of units in both wS2 and wS1 showed significant orientation sensitivity (Figures S7A and S7B) (all units with both horizontal and vertical stimulations pooled across ChR2 and eArch3.0 experiments: wS2: 32%, 218 of 680; wS1: 38%, 231 of 612).

During S2_L4_ inhibition, approximately 6% of wS2 units (30 of 467) showed an *OSI* value that was significantly modulated, with similar numbers of units exhibiting increased (12 of 30) and decreased (18 of 30) values of *OSI* (Figure 7D). In wS1, despite the modest effect of S2_L4_ inhibition on overall activity levels (Figure 6), we observed a significant modulation of orientation sensitivity in nearly 10% of units (37 of 398; Figures 7E–7H; corresponding to ~20% of the neurons in wS1 that were significantly orientation-sensitive). Almost all of the modulated units in wS1 (34 of 37) showed a reduction in orientation sensitivity (Figure 7H), indicating that under normal conditions (i.e., in the absence of inhibition) the effect of S2_L4_ activity is to enhance orientation tuning in wS1. The reduction in orientation sensitivity of wS1 units occurred rapidly, within the first 50 ms following onset of the whisker deflection (Figures S7C–S7E).

A reduction in orientation sensitivity can occur via a relative attenuation of the response to the preferred orientation and/or by a relative enhancement of the response to the non-preferred orientation. In wS2, changes in orientation sensitivity reflected a heterogeneous mixture of increases and decreases in responses to both the preferred and the non-preferred orientation (Figure 7I). In contrast, changes in orientation sensitivity in wS1 almost always involved a simultaneous reduction in the preferred-orientation response and an increase in the non-preferred-orientation response (Figures 7J and S7C–S7K). Inhibiting S2_L4_ therefore produced a coordinated and bidirectional response that acted to attenuate orientation tuning among a subset of neurons in wS1.

## DISCUSSION

Here, we probed the structure and dynamics of the S2 ↔ S1 circuit, using a combination of anatomical, electrophysiological, imaging, and optogenetic methods. Our data uncover key properties of a non-canonical feedback pathway that originates from layer 4 of wS2 and projects to wS1. This S2_L4_ → S1 pathway is rapidly activated by sensory stimuli, sends stimulus feature-specific information back to wS1 and modulates wS1 neurons in a manner that enhances orientation tuning in a subset of neurons in wS1.

The S2_L4_ → S1 feedback pathway we describe violates the canonical pattern whereby L4 neurons receive inputs from thalamus and lower cortical areas but do not send feedback projections back to lower areas (Felleman and Van Essen, 1991). This non-canonical feedback pathway acted to modulate activity in wS1, providing a basis for regulation of computations in primary sensory cortex.

Neurons labeled in L4 of rodent S2 have been seen before in studies using *trans*-synaptic viral (DeNardo et al., 2015) and conventional (Carvell and Simons, 1987; Fabri and Burton, 1991; Koralek et al., 1990) methods to retrogradely trace from S1. However, these S2_L4_ → S1 neurons have received remarkably little attention.

We found topographic specificity of the feedback projections from wS2 to wS1. Previous work in cats has shown somatotopic (homotypical) organization of cortico-cortical projections linking S2 and S1 (Alloway and Burton, 1985). Our goal was to determine whether such organization exists among whisker regions in rodents. Previous studies had shown that projections from wS1 to wS2 preserved somatotopy (Hoffer et al., 2003; Mao et al., 2011), and areas of wS2 and wS1 were reciprocally connected (Aronoff et al., 2010; Cauller et al., 1998; White and DeAmicis, 1977), implying that wS2 → wS1 feedback is somatotopic. Horizontal axons that bring top-down projections from multiple higher areas to L1 of rat wS1, however, extend over multiple barrel columns (Cauller et al., 1998), suggesting a lack of topographic specificity in the total top-down input to L1 at a location in wS1. Here, we used systematic injections of dual-color retrograde tracers, in addition to co-injection of an anterograde tracer, to provide a direct demonstration that feedback from wS2 back to wS1 is organized somatotopically. Whisker regions of S1 and S2 are therefore linked by “somatotopic loops,” such that information about stimulus location on the whisker array could be preserved as activity propagates in a loop between S1 and S2 (Kwon et al., 2016).

Touch-evoked activity occurred nearly as rapidly in wS2 as in wS1, including among the population of putative S2_L4_ → S1 neurons. We focused our analyses of timing on the mean response across large numbers of individual neurons. Across these large populations, the earliest responses began slightly (~2 ms) earlier in wS1 compared with wS2. This short delay is consistent with a monosynaptic delay, and thus with feedforward drive of wS2 neurons by wS1, but may reflect other factors such as differences in thalamocortical recruitment. Both areas receive direct thalamic input (Bosman et al., 2011; Carvell and Simons, 1987; Chakrabarti and Alloway, 2006; Feldmeyer, 2012; Koralek et al., 1988; Pierret et al., 2000). Regardless of their origins, our data show that wS2 responses, including those of putative S2_L4_ → S1 feedback neurons, occur rapidly following the onset of whisker deflections, at latencies approaching those of wS1 neurons (see also Kwegyir-Afful and Keller, 2004). Rapid computations may be critical for the sense of touch, a sensory modality that appears evolved for speed, with microsecond-level spike timing precision among primary afferents (Bale et al., 2015).

The S2_L4_ → S1 pathway was rapidly activated by whisker stimulation, and S2_L4_ inhibition rapidly reduced orientation sensitivity in wS1. However, the S2_L4_ → S1 pathway may also be involved in top-down modulations affecting wS1 at longer delays, such as the slow, choice-predictive depolarization seen in wS1 (Kwon et al., 2016; Sachidhanandam et al., 2013; Yang et al., 2016). Future work in mice performing quantitative behavioral tasks will be required to determine whether the S2_L4_ → S1 pathway plays a role in other forms of top-down modulation and impacts perceptual outcome.

S2 in rodents is generally considered a higher brain area compared with S1, with larger and more state-dependent receptive fields (Brett-Green et al., 2004; Carvell and Simons, 1986; Chen et al., 2016; Krubitzer et al., 1986; Kwegyir-Afful and Keller, 2004; Kwon et al., 2016; Suter and Shepherd, 2015). S2 also sends a major projection to L1 of S1, whereas S1 does not specifically target L1 of S2. This asymmetric pattern of L1 projections is a defining feature of hierarchical relationships in cortex and situates S2 at a higher level. Thus, we refer to the S2_L4_ → S1 projection as a “feedback” pathway because (1) it projects from wS2 to wS1, descending the cortical hierarchy as defined by response properties and L1 innervation, and (2) it modulates sensory responses of wS1 neurons. However, a long-standing question in comparative neurophysiology is to what extent S2 sensory responses are derived from direct thalamic input to S2, as opposed to feedforward inputs from S1 (for discussion, see Garraghty, 2007; Garraghty et al., 1991). An important direction for future work will be to determine whether activity propagating along the S2_L4_ → S1 pathway constitutes feedback in the sense of depending on the output of wS1, as opposed to the output of a parallel thalamocortical circuit.

We used optogenetic excitation and inhibition of S2_L4_ neurons to probe the synaptic impact these neurons have on the local wS2 circuit and at long-range in wS1. Prior work investigated the effect of activation and inhibition of L4 neurons within wS1 and found that the net effect of sustained L4 excitation was to excite L2/3 and inhibit L5 (Pluta et al., 2015). Here, we found that while the transient effect of exciting S2_L4_ was to excite superficial neurons, the net effect of sustained S2_L4_ excitation was to inhibit both superficial and deep neurons in wS2. Thus, although L4 neurons in both wS1 and wS2 are rapidly activated by whisker stimuli, our results suggest that the local functions played by L4 neurons in each area are distinct.

Optogenetic inhibition of S2_L4_ neurons resulted in widespread effects across wS2 and wS1. This was despite the fact that our inhibition of S2_L4_ was relatively modest, such that whisker stimuli could still excite these neurons. In wS2, inhibition of S2_L4_ resulted predominantly in inhibition of neurons in other layers, particularly superficial neurons. In wS1, inhibition of S2_L4_ produced only very weak inhibition of overall activity across the population. However, despite this weak effect on overall activity, inhibition of S2_L4_ neurons degraded orientation sensitivity in a subset of individual wS1 neurons (~20% of orientation sensitive units, or ~10% overall). Intriguingly, this occurred via simultaneous decreases in responses to the preferred orientation and increases in responses to the non-preferred orientation. Such coordinated, “push-pull” regulation may result from direct input from S2 both to inhibitory neurons of multiple types (Wall et al., 2016) and to excitatory neurons (DeNardo et al., 2015) within S1.

A limitation of the anatomy and *in vivo* physiology we report here is that it cannot determine the precise synaptic and circuit mechanisms by which S2_L4_ optogenetic manipulations exert their effects. S2_L4_ excitation and inhibition impacted activity broadly in wS2. Thus, non-L4 pathways to wS1, such as those from L2/3 and L6, could be modulated by S2_L4_ and play a role. Moreover, while S2_L4_ → S1 axons were found mainly in L5A, L2/3, and L1 of wS1, most of the wS1 units modulated by S2_L4_ excitation were in deep layers. This could reflect a contribution from non-L4 wS2 → wS1 pathways, or result from the strong synaptic connections linking superficial and deeper layers of wS1 (Hooks et al., 2011; Lefort et al., 2009). Further experiments are necessary to determine the mechanisms by which optogenetic modulations of S2_L4_ impact wS1.

Together, our results show that an L4-originating feedback pathway is rapidly activated by touch and that the S2_L4_ neurons that form this pathway act to enhance the stimulus feature tuning of neurons in primary sensory cortex. Given the strong modulation of wS2 neurons and of their feedback to wS1 during perceptual decisions (Chen et al., 2016; Kwon et al., 2016; Yang et al., 2016; Zuo et al., 2015), our data suggest that the S2_L4_ → S1 pathway may regulate wS1 feature sensitivity during behavior.

## STAR★METHODS

Detailed methods are provided in the online version of this paper and include the following:

### KEY RESOURCES TABLE

**Table:** 

REAGENT or RESOURCE	SOURCE	IDENTIFIER
Antibodies		
Chicken anti-GFP polyclonal antibody	ThermoFisher Scientific	Cat#: A10262; RRID: AB_2534023
Rabbit anti-RFP polyclonal antibody	Rockland	Cat#: 600-401-379; RRID: AB_2209751
Mouse anti-GAD67 monoclonal antibody	Millipore	Cat#: MAB5406; RRID: AB_2278725
Chicken anti-GFP polyclonal antibody	Aves	Cat#: GFP-1020; RRID: AB_10000240
Rabbit anti-DsRed polyclonal antibody	Clontech	Cat#: 632496; RRID: AB_10013483
Bacterial and Virus Strains		
rAAV1/CAG-Flex-GFP	UNC Gene Therapy Center Vector Core	N/A
rAAV1/CAG-Flex-tdTomato	UNC Gene Therapy Center Vector Core	N/A
rAAV5.EF1a-DIO-hChR2(H134R)-EYFP	UNC Gene Therapy Center Vector Core	N/A
rAAV5/Ef1a-DIO-eArch3.0-EYFP	UNC Gene Therapy Center Vector Core	N/A
AAV1.CB7.CI.mCherry.WPRE.rBG	University of Pennsylvania Gene Therapy Program Vector Core	Cat#: AV-1-PV1969
AAV1.CB7.CI.eGFP.WPRE.rBG	University of Pennsylvania Gene Therapy Program Vector Core	Cat#: AV-1-PV1963
AAV1.CAG.FLEX.GCaMP6s.WPRE.SV40	University of Pennsylvania Gene Therapy Program Vector Core	Cat#: AV-1-PV2818
rAAV2-retro-Syn-Cre	HHMI Janelia Virus Service Facility	Tervo et al., 2016
Chemicals, Peptides, and Recombinant Proteins		
CTB-Alexa488	ThermoFisher Scientific	Cat#: C34775
CTB-Alexa555	ThermoFisher Scientific	Cat#: C34776
CTB-Alexa647	ThermoFisher Scientific	Cat#: C34778
Experimental Models: Organisms/Strains		
Mouse: C57BL/6NHsd	Envigo International Holdings	Cat#: 004
Mouse: B6;C3-Tg(Scnn1a-cre)3Aibs/J	The Jackson Laboratory	JAX: 009613
Mouse: B6;129S-Gt(ROSA)26Sor^tm32(CAG-COP4^*^H134R/EYFP)Hze^/J	The Jackson Laboratory	JAX: 012569
Mouse: B6.Cg-Gt(ROSA)26Sor^tm9(CAG-tdTomato)Hze^/J	The Jackson Laboratory	JAX: 007909
Mouse: RORβ-GFP	Liu et al., 2013	MGI: 5548299
Software and Algorithms		
MATLAB versions R2014a, R2016b and R2017a	MathWorks	RRID: SCR_001622
Kilosort	https://github.com/cortex-lab/Kilosort	N/A
WaveSurfer	HHMI Janelia Research Campus	http://wavesurfer.janelia.org
Ephus	Suter et al., 2010; Vidrio Technologies	http://scanimage.vidriotechnologies.com/display/ephus/Ephus
ScanImage version 4.2	Vidrio Technologies	http://scanimage.vidriotechnologies.com/display/SIH

### CONTACT FOR REAGENT AND RESOURCE SHARING

Further information and requests for resources and reagents should be directed to and will be fulfilled by the Lead Contact, Daniel H. O’Connor (dan.oconnor@jhmi.edu).

### EXPERIMENTAL MODEL AND SUBJECT DETAILS

All procedures were in accordance with protocols approved by the Johns Hopkins University Animal Care and Use Committee. All *in vivo* electrophysiology and calcium imaging experiments were done in awake, head-fixed mice.

#### Mice

We report anatomical experiments in 47 C57BL/6NHsd (Harlan) mice; 2 mice obtained by crossing Scnn1a-Tg3-Cre (Madisen et al., 2010; Jackson Labs: 009613; B6;C3-Tg(Scnn1a-cre)3Aibs/J) mice with Ai32 (Madisen et al., 2012; Jackson Labs: 012569; B6;129S-Gt(ROSA)26Sor^tm32(CAG-COP4^*^H134R/EYFP)Hze^/J) mice on a mixed background; 5 mice obtained by crossing Scnn1a-Tg3-Cre mice with Ai9 (Madisen et al., 2010; Jackson Labs: 007909; B6.Cg-Gt(ROSA)26Sor^tm9(CAG-tdTomato)Hze^/J) mice; 2 Ai9 mice; one heterozygous RORβ-GFP (Liu et al., 2013) mouse (gift of S. Nelson, Brandeis University); 16 Scnn1a-Tg3-Cre mice; tetrode recordings in 10 C57BL/6NHsd mice from S1; 15 C57BL/6NHsd mice and 6 Scnn1a-Tg3-Cre mice from S2; silicon probe recordings in 13 and 12 Scnn1a-Tg3-Cre mice from S1 and S2, respectively. We report two-photon imaging experiments from 4 Scnn1a-Tg3-Cre mice. All mice were male. Ages ranged from 5–15 weeks as described below. Mice were housed in a vivarium with reverse light-dark cycle (12 h each phase). Experiments occurred during the dark phase. Mice were housed in groups of up to 5 unless the animals were used for electrophysiology or imaging studies. For these electrophysiology and imaging studies, animals were housed singly after implantation of tetrodes or cranial window, or for 2 weeks prior to acute silicon probe recordings. Mouse cages included enrichment materials such as bedding and plastic domes (Innodome, Innovive).

### METHOD DETAILS

#### Adeno-associated viruses

We obtained the following AAV viruses from the UNC Gene Therapy Center Vector Core: rAAV1/CAG-Flex-GFP (abbreviated elsewhere in this manuscript as “AAV-Flex-GFP”), rAAV1/CAG-Flex-tdTomato (“AAV-Flex-tdTomato”), rAAV5.EF1a-DIO-hChR2(H134R)-EYFP (“AAV-DIO-hChR2-EFGP”), rAAV5/Ef1a-DIO-eArch3.0-EYFP (“AAV-DIO-eArch3.0-EYFP”). We obtained the following from the University of Pennsylvania Gene Therapy Program Vector Core: AAV1.CB7.CI.mCherry.WPRE.rBG (“AAV-XFP,” or “AAV-mCherry”), AAV1.CB7.CI.eGFP.WPRE.rBG (also “AAV-XFP,” or “AAV-EGFP”), AAV1.CAG.FLEX.GCaMP6s.WPRE.SV40 (“AAV-Flex-GCaMP6s”). We obtained rAAV2-retro-Syn-Cre (Figure S3) from the Janelia Virus Service Facility.

#### Headpost implantation

Titanium headposts were implanted for head fixation (O’Connor et al., 2013) at 5–7 weeks of age. Briefly, mice were anesthetized (1%–2% isoflurane in O_2_; Surgivet) and mounted in a stereotaxic apparatus (David Kopf Instruments). Body temperature was maintained with a thermal blanket (Harvard Apparatus). The scalp and periosteum over the dorsal surface of the skull were removed. The skull surface over the posterior half of the left hemisphere, which covers S1 and S2, was thinned with a dental drill. The remaining exposed area of the skull was scored with a dental drill and the head post affixed using cyanoacrylate adhesive (Krazy Glue) followed by dental acrylic (Jet Repair Acrylic). An opening (“well”) in the head post over the left hemisphere was covered with silicone elastomer (Kwik-Cast, WPI) followed by a thin layer of dental acrylic.

#### Intrinsic signal imaging

After recovery from headpost surgery (> 24 h), mice were lightly anesthetized with isoflurane (0.5%–1%) and chlorprothixene (0.02 mL of 0.36 mg mL^–1^, intramuscular). Intrinsic signal imaging (ISI) was performed as described (O’Connor et al., 2013). In most cases, the target whisker was right D2. In rare cases D2 was missing at the time of ISI, and D1 or D3 was substituted. ISI was performed through the skull covered by a thin layer of cyanoacrylate adhesive. Whisker S2 could be identified as a region of decreased reflectance clearly delineated from whisker S1 (Kwon et al., 2016). Sound from the piezo stimulator is a potential source of response during ISI mapping of regions in the vicinity of S2. To distinguish auditory responses from tactile responses, ISI was occasionally performed without threading the target whisker into the stimulator, which otherwise remained in a nearly identical position. Areas responsive under this condition were considered auditory areas and were distinct from whisker S2. These auditory responses were almost completely masked by a constant, white-noise like sound via an aspirator placed adjacent to the stimulated whisker. In some animals, two colors of CTB-Alexa were co-injected into S1 and S2 according to ISI signals (see below). In those experiments, retrogradely labeled cells from S1 were localized around the center of the S2 injection, serving as a validation of the ISI-guided identification of S2. When CTB-Alexa injections were replaced with AAV-XFP injections, dense S1 axonal fibers overlapped with the S2 injection site.

#### Anatomical tracer and virus injections

CTB and AAV were injected via small craniotomies over S1 or S2 localized by intrinsic signal imaging, using a beveled glass pipette (30–50 μm ID), in 6–8 week old mice unless otherwise specified. In most animals, injections (rate: 1 nL s^–1^) were done at 4 different depths (from the pia: 300, 400, 600, 800 μm in S1; 300, 500, 700, 900 μm in S2) with the goal of covering the full thickness of cortex without spreading into the white matter. For labeling of L4 neurons in Scnn1a-Tg3-Cre mice, AAVs were injected at 2 depths (300, 500 μm in S1; 400, 600 μm in S2) from the pia. Injections were carried out mostly at 3–5 weeks of age in Scnn1a-Tg3-Cre mice for efficient labeling. After the injection, the well in the head post was sealed with silicone elastomer and dental acrylic as described above.

For retrograde labeling, mice were injected with retrograde tracer conjugated with three types of fluorescent Alexa dyes (CTB-Alexa488, CTB-Alexa555 and CTB-Alexa647, 5 μg μL^–1^ in PBS, ThermoFisher Scientific). 30 nL of CTB-Alexa was injected at each depth (120 nL in total). rAAV2-retro-Syn-Cre was also used for retrograde labeling of cell bodies (Figure S3) via injection in Ai9 mice (7.5 nL at each depth, 30 nL in total). Anterograde labeling of axons was done via injection of AAV-XFPs (10 nL at each depth, 40 nL in total). Co-injection of CTB-Alexa and AAV-EGFP was via a cocktail with a CTB-Alexa:AAV-EGFP ratio of 3:1 (40 nL at each depth, 160 nL in total). L4 neurons were labeled by injection of AAV-Flex-GFP or AAV-Flex-tdTomato in Scnn1a-Tg3-Cre mice at 2 depths (15 nL at each depth, 30 nL in total for Figures S4A–S4C; 100 nL at each depth, 200 nL in total for Figure S2B). For identification of morphology, S2_L4_ neurons were sparsely labeled with AAV-Flex-tdTomato diluted 10-fold with PBS (100 nL at each depth, 200 nL in total). Anterograde labeling of thalamocortical axons originating from the posterior medial nucleus of the thalamus (POm) was done via injection of AAV-EGFP (20 nL at stereotaxic coordinates: 1350 μm posterior, 1180 μm lateral, 3300 μm ventral from Bregma) in Scnn1a-Tg3-Cre;Ai9 mice at 7 weeks of age. For anatomical studies, the animal was sacrificed 1 to 2 weeks (CTB), 1 week (anterograde labeling of POm axons with AAV) or 2 to 3 weeks (other AAV experiments) after the injection, and the brain processed (see “Tissue processing” section below).

For physiological experiments, AAV-DIO-hChR2-EFGP or AAV-DIO-eArch3.0-EYFP were injected at each of two depths (40–60 nL at each depth, 80–120 nL total) in Scnn1a-Tg3-Cre mice. Physiological experiments were performed 3–5 weeks (AAV-DIO-hChR2-EFGP) or 5–8 weeks (AAV-DIO-eArch3.0-EYFP) after the injection.

#### Tissue processing

Mice were perfused transcardially with 4% paraformaldehyde in 0.1 M PB (4% PFA). Brains were sectioned coronally at a thickness of 80–100 μm after overnight (10–12 hr) post-fixation in 4% PFA, except where specified. GFP and tdTomato signals were amplified by immunostaining with chicken anti-GFP (1:500; A10262, ThermoFisher Scientific) and rabbit anti-RFP (1:500; 600-401-379, Rockland). To aid visualization of cortical layer boundaries and barrels, sections were stained by mouse anti-GAD67 (1:500; MAB5406, Millipore; Meyer et al., 2010) after 4–5 hr (rather than overnight) post-fixation.

For experiments in Figures 2, S5E, and S5F, we made tangential instead of coronal sections. After perfusion, cortical blocks containing S1 and S2 were flattened between microscope slides and post-fixed for 4–5 hr in 4% PFA. The flattened tissue was sectioned parallel to the pia at a thickness of 100 μm.

Sections were mounted on glass slides in DAPI-containing mounting medium (H-1200, Vector Laboratories). Images of processed tissues were acquired via CCD camera (QImaging, QIClick) and epifluorescence imaging (BX-41, Olympus), or with a confocal microscope (LSM 510, Zeiss).

For experiments labeling thalamocortical axons from POm (Figures 1E–1H and S1E–S1L), we made 60 μm coronal sections and stained to amplify EGFP (1:2000 chicken anti-GFP; GFP-1020, Aves) and tdTomato (1:2000 rabbit anti-DsRed; 632496, Clontech Laboratories). Sections were then mounted using Aqua Poly/Mount mounting medium (Polysciences, Inc) and imaged on a confocal microscope (LSM 800, Zeiss) using a 10x (0.3 NA) objective. Brightness and contrast were adjusted using Adobe Photoshop (Adobe Systems).

#### Cortical layer boundaries

L1-to-L2/3 and L6-to-white matter boundaries are clear from autofluorescence and DAPI staining. To further estimate layer boundaries, coronal sections were stained for GAD67 to yield two bands of dense labeling in both S1 and S2. The upper band in S1 has a barrel-like structure that overlaps well with the barrels delineated from Cre-dependent EYFP signal in Scnn1a-Tg3-Cre;Ai32 mice. This upper band extends to S2 and again shows a clear overlap with the EYFP signal from Scnn1a-Tg3-Cre;Ai32 mice, and with GFP fluorescence in a RORβ-GFP mouse (Figure S1). Therefore, we used GAD67 as an L4 marker in S2 and Scnn1a-Tg3-Cre mice for labeling S2_L4_ neurons. The lower band of GAD67 labeling was used as a marker of the L5A-to-L5B border (Meyer et al., 2010).

#### Cortical depth normalization

“Normalized depth” (Figures 1C, 1D, S3C, and S4C) within cortex was defined separately for each animal with respect to the pial surface and the white matter. To plot layer boundaries for group data, we used the population averages of normalized layer boundary depths. The upper boundaries of L2/3, L4, L5A, L5B, L6 were as follows: 0.081 ± 0.019, 0.279 ± 0.028, 0.440 ± 0.039, 0.538 ± 0.032, 0.651 ± 0.026 in S1 (mean ± SD; 34 sections from 25 mice); 0.080 ± 0.022, 0.310 ± 0.028, 0.433 ± 0.024, 0.521 ± 0.022, 0.621 ± 0.022 in S2 (28 sections from 23 mice).

#### Axonal fluorescence normalization

To normalize axonal fluorescence (Figures 1C, 1D, and S4C), the angle of each histological image was adjusted such that a region of interest (ROI) spanning from pia to white matter was horizontal. A second, control region was chosen for each section based on lack of evident axonal fibers. The mean pixel value of this control region was used to determined background fluorescence. Within the ROI, the mean fluorescence for each row of pixels (spanning the full depth) was calculated. From these mean values we subtracted the background fluorescence obtained from the control region, then divided by the grand mean of the background-subtracted fluorescence averaged over depth from pia to the white matter within the ROI. The resulting quantity we refer to as “normalized fluorescence.”

#### Electrophysiology

We recorded from multiple single units simultaneously using tetrodes or silicon probes in mice aged 9–15 weeks. Tetrode recordings were carried out using a custom-built screw-driven microdrive with eight implanted tetrodes (32 channels total; Cohen et al., 2012). Tetrodes were implanted perpendicular to the cortical surface, which for wS1 and wS2 are ~30 degrees and ~55 degrees from vertical, respectively. Neural signals were filtered between 0.09 Hz and 7.6 kHz, and together with time stamps for whisker or optogenetic stimuli, were digitized and recorded continuously at 20 kHz (RHD2000 system with RHD2132 headstage, Intan Technologies). Spike waveforms were extracted by thresholding on bandpass filtered (700–6,000 Hz) signals and sorted offline using custom software. To measure unit isolation quality, we calculated the L-ratio (Schmitzer-Torbert and Redish, 2004) and the fraction of inter-spike interval (ISI) violations for a 2 ms refractory period. All units included in the dataset had L-ratios < 0.05 and < 1% ISI violations. Because application of these two criteria require a sufficient number of spikes, we included only units with overall spike frequencies > 1 Hz, which corresponded to 2,000–3,000 spikes given the 40–50 min recording session durations (spikes from periods of photostimulation that began after the main recording session were not included). Recording sites were verified histologically using electrolytic lesions (15 s of 10 mA direct current).

Silicon probe recordings were via a 64-channel linear probe (ASSY-77 H3, Cambridge NeuroTech). Recordings were done up to three times from an area with the same angle and depth but with slightly (50–100 μm) different positions in tangential plane. The probe was coated with DiI to histologically verify the recording area and to reconstruct the depths of channels within cortex. The probe was inserted into cortex at ~30 degrees and ~45 degrees from vertical for S1 and S2 recordings, respectively. The probe was left for 30 min before recordings for tissue relaxation. Neural signals were filtered between 0.09 Hz and 7.6 kHz, and together with time stamps for whisker or optogenetic stimuli, were digitized and recorded continuously at 30 kHz (RHD2000 system with RHD2164 headstage, Intan Technologies). Spike sorting was carried out with Kilosort (http://biorxiv.org/lookup/doi/10.1101/061481). We included neurons with < 1% ISI violations of a 2 ms refractory period. We excluded units that spiked on < 95% of the stimulus trials within a session, or with unstable spike shapes assessed by visual inspection. For reconstruction of channel positions within the cortex, the DiI-marked point of the deepest probe tip insertion was located with respect to cortical layer boundaries, estimated using GAD67 or EYFP signals (described in the “Cortical layer boundaries” section). Additionally, we used whisker-evoked current source density (CSD; “CSDPlotter” package in MATLAB) analysis to estimate the middle of L4 for each recording. The probe location corresponding to the middle of L4, together with the histological determination of probe insertion depth within cortex, allowed us to assign the laminar location of each channel as “L4,” “superficial” (above L4) or “deep” (below L4).

#### Optogenetics

For tetrode recordings in wS2 combined with axonal photostimulation of S2_L4_→S1 neurons, a 200-μm, 0.39 NA optic fiber was co-implanted with the tetrode microdrive such that its tip was < 0.5 mm above the surface of the D2 barrel column (targeted by ISI) in S1. The optic fiber was coupled to a 473 nm laser (DHOM-L-473-200mW, UltraLasers) with intensity controlled by an acousto-optic modulator (MTS110-A3-VIS, QuantaTech). After each recording session, trains of 20 light pulses (2 ms pulse duration, 10 mW from the fiber ending) were delivered at 20 Hz. These light pulse trains were repeated for 15 cycles (300 pulses total). Based on estimates of light spread in brain tissue (Yizhar et al., 2011; Yona et al., 2016), our photostimulus is expected to permit optogenetic excitation in a volume limited to S1. Putative antidromically activated S2_L4_→S1 single units were assigned based on latency and reliability of responses to this photostimulation. First, the latency of light-evoked activity was estimated. Spikes occurring within a range of [−25, 25 ms] from each of the 300 light pulses were collected and used to construct a peri-stimulus time histogram with 1 ms bin size. The three consecutive bins with the maximum number of spikes within the 25 ms period following light pulse onset was determined. The latency of the response was calculated as the mean latency of all spikes falling within these three bins (Zhang et al., 2013). Second, we quantified reliability of the photo-evoked response. Spikes occurring at the determined latency ± 1.5 ms relative to the onset of each light pulse were counted separately for the first to the twentieth light pulse within a train (summing across all 15 repetitions of the stimulus pulse train). These 20 spike counts were compared with baseline activity. A spike count histogram with 3 ms bin size was constructed for the range of [−999, 0 ms] relative to the onset of the light pulse train. If a light-evoked spike count exceeded mean + 1.96 SD of these binned baseline activity values, the response was regarded to be “significant” for that particular light pulse within the 20-pulse sequence. Neurons that showed at least 18 significant responses out of the 20 total pulses were considered to respond reliability and assigned as putative S2_L4_→S1 units.

For somatic excitation of S2_L4_ neurons simultaneous with whisker stimulation during silicon probe recordings, the tip of a 400 μm, 0.39 NA optic fiber (Thorlabs) was placed ~2 mm above the surface of S2. The optic fiber was coupled to a 470 nm LED (M470F3, Thorlabs). Constant light (3.5–12.5 mW) was applied during an interval of [−100, 500 ms] from whisker stimulus onset, with additional 100 ms ramp-up and ramp-down periods.

Somatic inhibition of S2_L4_ was conducted with the same configuration, except using a 565 nm LED (M565F3, Thorlabs) and longer periods of light delivery. In 11 recordings from 4 mice, light delivery spanned [−100, 500 ms] from whisker stimulus onset with additional 100-ms ramp-up and ramp-down periods. In 3 recordings from 2 mice, light delivery spanned [−500, 500 ms] with additional 100-ms ramp-up and ramp-down periods. In 2 recordings from 1 mouse, light delivery spanned [−500, 500 ms] with no ramp-up or ramp-down periods. Light power during the constant phase was 12.5 mW. Trials with and without optogenetic stimuli were interleaved as described below.

#### Whisker stimulation

All whiskers except the target whisker were trimmed to near the base. The target whisker was threaded into a glass pipette attached to a 2D piezo actuator (NAC2710-A01, Noliac) or a 1D piezo actuator (Q220-A4-203YB or D220-A4-203YB, Piezo Systems), with ~3–5 mm at the base exposed. The whisker was deflected for 0.5 s with a 40 Hz sinusoidal deflection, using a piezo driver (MDT693A or MDTC93B, Thorlabs) and Ephus (Suter et al., 2010) or WaveSurfer (http://wavesurfer.janelia.org). For single-orientation stimuli, the deflection was rostral-caudal (~800 deg/s), with trials starting every 4.5 s (i.e., a new deflection waveform started every 4.5 s). For two-orientation stimuli, the deflections (~1,020 deg/s) were either rostral-caudal (horizontal) or dorsal-ventral (vertical), with trials starting every 3.5 s.

For electrophysiology experiments, two-orientation stimuli were given in the following sequence of trials: horizontal → horizontal → vertical → vertical. Optogenetic stimulation occurred during the first and third of these trials. The sequence was repeated 120 times (480 deflections total). The stimulated whisker was typically right D2, as described in the “Intrinsic signal imaging” section.

For two-photon imaging experiments, the stimulus orientation was altered in a randomly interleaved manner, subject to the constraint that no more than 3 in a row of the same type were delivered. The right C2 whisker was used in 2 mice and right D3 in 2 mice.

#### Two-photon calcium imaging

A titanium headpost was implanted on P30–35 mice, and 2 days later intrinsic signal imaging performed to localize wS1 and wS2, as described above. GCaMP6s (Chen et al., 2013) was expressed in a Cre-dependent manner under the CAG promoter following infection with AAV1.CAG.FLEX.GCaMP6s.WPRE.SV40. In P35–40 mice (5 days after head plate implantation) under light isoflurane (1%–1.5%), the skull above the C2 or D3 area within wS2, previously localized via intrinsic signal imaging, was thinned with a dental drill. A craniotomy (~200 μm diameter) was made by removing a small piece of the thinned bone with a tungsten needle (Fine Science Tools). The dura was left intact. Injections were made at 2 depths within the craniotomy (50 nL per depth; depths, 500 μm and 400 μm; rate, ~1 nL per second) using a glass pipette (30–50 μm diameter). After injection, the pipette was left in place for 3 min, and the craniotomy was covered with dental cement. A large circular craniotomy (2.5 mm diameter) was made over the C2 or D3 barrel area, localized via intrinsic signal imaging, within wS1. This craniotomy was placed ~1.5 mm off of the barrel center (medially) to avoid an overlap with the wS2 injection site. An imaging window was made by gluing together two pieces of microscope cover glass (Huber et al., 2012). The smaller piece (Fisher; number 2 thickness) was fitted into the craniotomy and the larger piece (number 1.5 thickness) was glued to the bone surrounding the craniotomy. Whiskers other than the relevant whisker on the right side of the snout were shortened at the time of intrinsic signal imaging (to ~1 cm) to aid single-whisker stimulation, then allowed to regrow until the day of the imaging session 14–20 days later.

Images were acquired on a custom-built two-photon microscope (http://openwiki.janelia.org/wiki/display/shareddesigns/MIMMS) equipped with a resonant scanning module (Thorlabs), GaAsP photomultiplier tubes (Hamamatsu) and a 16x 0.8 NA microscope objective (Nikon). GCaMP6s was excited at 960 nm with a Ti:Sapphire laser (Chameleon Ultra II, Coherent). Imaging fields were restricted to areas where S2_L4_→S1 axons expressing GCaMP6s overlapped with the desired barrel columns. Imaging depth ranged from 60 μm to 160 μm (layer 1–2). The field of view was 273 μm x 298 μm (440 × 512 pixels; pixel size, 0.62 μm x 0.58 μm).

Images were acquired continuously at 30 Hz using ScanImage (Pologruto et al., 2003) version 4.2 (http://scanimage.vidriotechnologies.com). A single trial comprised 140 image frames. Mice were awake during image acquisition. For each mouse, multiple fields of view (2–5 different depths, 60–160 μm from the pial surface at the same lateral position) were acquired.

#### Data analysis: calcium imaging

A line-by-line correction algorithm (Peron et al., 2015) was used to correct for brain motion. For each trial we used five consecutive frames with a minimum of luminance changes to generate an average reference image. Each line was registered to the reference image by maximizing the line-by-line Pearson correlation. Regions of interest (ROIs) corresponding to individual boutons were manually selected. For each ROI, the time series of raw fluorescence was estimated by averaging all pixels within the ROI. ΔF/F_0_ was calculated as (F-F_0_)/F_0_, where F_0_ was the mean F over 6 baseline frames preceding the stimulus onset time (between frames 52 and 53) for each trial. Evoked ΔF/F_0_ was calculated as the mean ΔF/F_0_ over 6 frames following the stimulus onset time (frames 55–60 with stimulus onset between frames 52 and 53). Mean vertical and horizontal evoked ΔF/F_0_ values were calculated by averaging evoked ΔF/F_0_ values on vertical or horizontal stimulus trials, respectively.

We used receiver operating characteristic (ROC) analysis to define vertically or horizontally tuned ROIs (Figure 4). A decision variable (DV) was assigned for each trial based on the neural response. DV was the mean evoked ΔF/F_0_ as defined above. Trials were grouped by stimulus condition (vertical versus horizontal deflections). An ROC curve was obtained by systematically varying the criterion value across the full range of DV (using MATLAB “perfcurve”). The area under the ROC curve (AUC) represents the performance of an ideal observer in categorizing trials based on the DV. A 95% confidence interval for each AUC was obtained by bootstrap (MATLAB “perfcurve”). ROIs with AUC significantly larger than 0.5 were defined as vertically tuned, and those with AUC significantly smaller than 0.5 as horizontally tuned.

#### Data analysis: electrophysiology

After spike sorting, we excluded from further analysis outlier trials typically associated with large animal movements. Outlier trials were defined as those with a spike count beyond 3 standard deviations from the mean, calculated using all trials within the same stimulus category (i.e., stimulus orientation and presence/absence of an optogenetic stimulus). In experiments with two-orientations of whisker stimulation and either presence or absence of optogenetic stimulation, the four categories of trial defined by these conditions were presented sequentially and defined a trial “cycle.” When an outlier trial was detected in a cycle, the other three trials within the same cycle were also excluded.

For the mean wS1 and wS2 PSTHs in Figure 3G only, in order to obtain as much statistical power as possible to resolve latency differences between wS1 and wS2 responses to the whisker stimulus, we combined data from tetrode recordings and from the silicon probe recordings reported in Figures 5, 6, and 7. For our tetrode recordings, we delivered rostral-caudal deflections only, as described above. For silicon probe recordings, we delivered both rostral-caudal and dorsal-ventral deflections. To pool data for Figure 3G across similar conditions, we included only trials with rostral-caudal deflections from the silicon probe recordings, and only trials without optogenetic stimulation.

The F1/F0 value (Figures 3H–3J) used to quantify the degree to which a unit’s spike rate was modulated by the sinusoidal whisker deflection stimulus on a cycle-by-cycle basis was obtained after kernel density estimation (MATLAB “ksdensity”) of the stimulus-aligned spike rate. F1 was the range of this density estimate, and F0 was the mean.

The optogenetic modulation index for a given time window was calculated as: (*R_Stim_* − *R_Nostim_*)/(*R_Stim_* + *R_Nostim_*), where *R_Stim_* and *R_Nostim_* indicate firing rates with and without optogenetic stimulation. Although whisker deflections were given along two orientations for the experiments shown in Figures 5 and 6, stimulus orientation was not taken into account for calculation of the optogenetic modulation index. In boxplots in Figure 6H, optogenetic modulation index values greater than q_3_+w × (q_3_−q_1_) or less than q_1_−w × (q_3_−q_1_) were considered outliers and not plotted, where q_1_ and q_3_ are the first and third quartiles and w = 1.5.

The orientation sensitivity index (*OSI*) was calculated as: *OSI* = (*R_Pref_* – *R_Nonpref_* )/(*R_Pref_* + *R_Nonpref_* ), where *R_Pref_* and *R_Nonpref_* indicate firing rates during periods of whisker stimulation with preferred and non-preferred orientations. We defined the preferred orientation of a unit as the orientation of the whisker stimulus that evoked the larger number of spikes during the 500 ms stimulus period, averaged across the recording session.

### QUANTIFICATION AND STATISTICAL ANALYSIS

Data analyses were conducted in MATLAB. Data are reported as mean ± SEM unless otherwise noted. All statistical tests were two-tailed. A unit was regarded as “whisker responsive” when its spike rate on trials without optogenetic perturbation was significantly higher (p < 0.01 by Sign test for paired samples, MATLAB “signtest”) during the period of the 500 ms stimulus compared with the immediately preceding 500 ms period. Units inhibited by whisker stimulation (~10% in both wS1 and wS2) were thus not considered whisker responsive. PSTHs in Figure 3G were limited to those wS1, wS2 and S2_L4_→S1 units that were excited by the whisker stimulus within the first 50 ms after stimulus onset, defined as having a spike rate that was significantly higher (p < 0.01 by Sign test for paired samples, MATLAB “signtest”) during the period of the first 50 ms following stimulus onset compared with the immediately preceding 50 ms period.

We considered a unit orientation sensitive when its spike rates during the period of the 500 ms stimulus differed for horizontal and vertical whisker deflections (p < 0.01 by Sign test for paired samples, MATLAB “signtest”).

We used a bootstrap method to test the significance of changes in *OSI* resulting from optogenetic inhibition of S2_L4_ (Figures 7D and 7H). The test statistic was the difference in *OSI* calculated as described above for inhibited (LED ON) and control (LED OFF) conditions, Δ*OSI* = *OSI*_ON_ – *OSI*_OFF_. On each of 5,000 iterations, we calculated a bootstrap replicate, ΔOSI*. Each replicate was obtained by sampling trials randomly with replacement separately for each of the four conditions (horizontal/vertical whisker stimulus X LED ON/OFF), to obtain four new bootstrap samples, each the same size as the original. ΔOSI* was then calculated based on these bootstrap samples. A 95% confidence interval for Δ*OSI* was obtained using the 2.5^th^ and 97.5^th^ percentiles of the distribution of ΔOSI*. When this confidence interval did not include zero, the unit was considered to show a significant Δ*OSI*.

### DATA AND SOFTWARE AVAILABILITY

Custom MATLAB code used for analyses and data will be made available upon reasonable request.

## Supplementary Material

1

2

## Highlights

- Layer 4 of S2 sends a major feedback pathway to S1
- S2 to S1 feedback is somatotopic within the whisker zones
- S2 layer 4 feedback is orientation specific and rapidly activated
- S2 layer 4 feedback modulates activity and orientation tuning in S1
