# Supplementary material for: A Non-canonical Feedback Circuit for Rapid Interactions between Somatosensory Cortices
Source: Cell Rep. Author manuscript; Available in PMC 2018 Jun 18. (PMC6004823; doi:10.1016/j.celrep.2018.04.115)
Supplement: 1 [file NIHMS973281-supplement-1.pdf]

**Cell Reports, Volume *23***

## Supplemental Information

# A Non-canonical Feedback Circuit

# for Rapid Interactions

# between Somatosensory Cortices

**Genki Minamisawa, Sung Eun Kwon, Maxime Chevée, Solange P. Brown, and Daniel H. O'Connor**

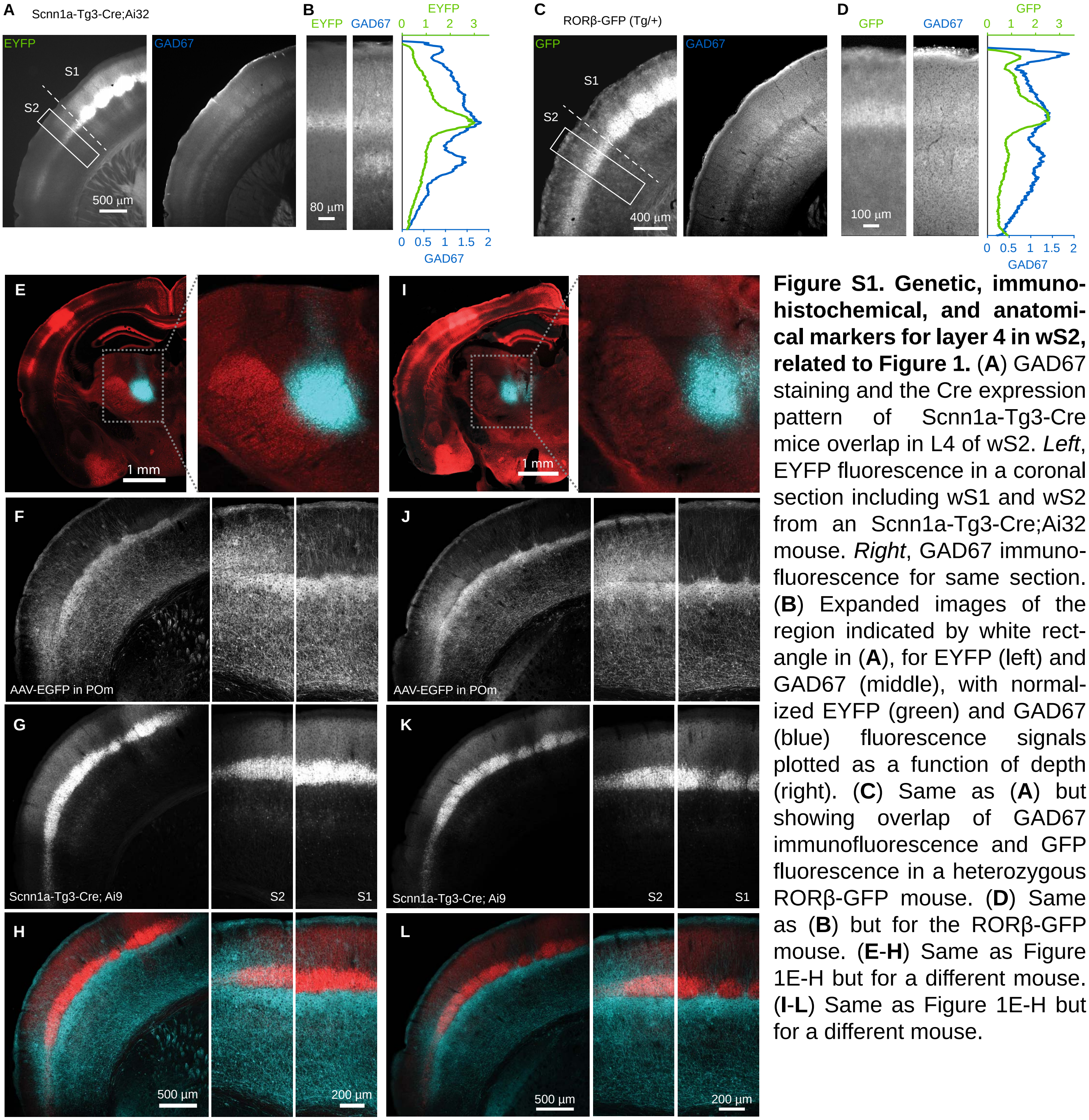


**Figure S1. Genetic, immunohistochemical, and anatomical markers for layer 4 in wS2, related to Figure 1.** (**A**) GAD67 staining and the Cre expression pattern of Scnn1a-Tg3-Cre mice overlap in L4 of wS2. *Left*, EYFP fluorescence in a coronal section including wS1 and wS2 from an Scnn1a-Tg3-Cre;Ai32 mouse. *Right*, GAD67 immunofluorescence for same section. (**B**) Expanded images of the region indicated by white rectangle in (**A**), for EYFP (left) and GAD67 (middle), with normalized EYFP (green) and GAD67 (blue) fluorescence signals plotted as a function of depth (right). (**C**) Same as (**A**) but showing overlap of GAD67 immunofluorescence and GFP fluorescence in a heterozygous RORβ-GFP mouse. (**D**) Same as (**B**) but for the RORβ-GFP mouse. (**E**-**H**) Same as Figure 1E-H but for a different mouse. (**I**-**L**) Same as Figure 1E-H but for a different mouse.

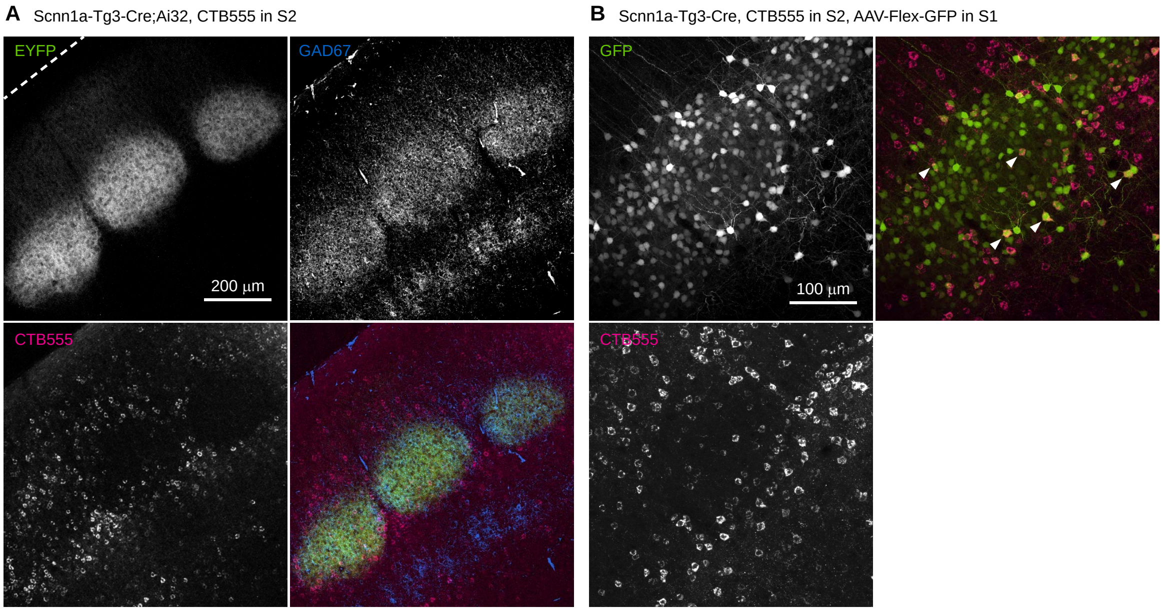


**Figure S2. S1→S2 neurons in the regions surrounding barrels, related to Figure 1.** (**A**) S1→S2 neurons are mostly outside of barrels. *Top left*, ChR2-EYFP fluorescence showing barrels in wS1 from an Scnn1a-Tg3-Cre;Ai32 mouse. *Top right*, GAD67 immunofluorescence for same field of view. *Bottom left*, S1→S2 neurons are retrogradely labeled with CTB-Alexa555, same field of view. *Bottom right*, Overlay of EFYP, GAD67 and CTB-Alexa555 images. (**B**) Similar to (**A**), but with cytosolic GFP fluorescence instead of ChR2-EFYP. *Top left*, GFP fluorescence from wS1 of an Scnn1a-Tg3-Cre mouse injected with an AAV virus (AAV-Flex-GFP) that gives Cre-dependent expression of GFP. *Bottom left*, S1→S2 neurons are retrogradely labeled with CTB-Alexa555, same FOV. *Top right*, Overlay of GFP and CTB-Alexa555 images. Double labeled neurons (white arrowheads) occur but are relatively few. (**A**-**B**) Images are confocal stack z-projections.

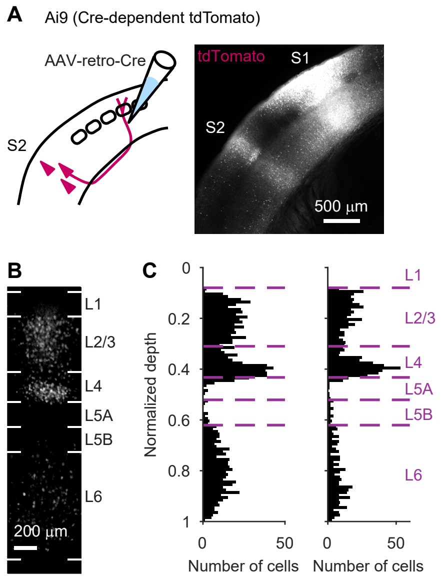


**Figure S3. S2→S1 neurons retrogradely labeled using rAAV2-retro, related to Figure 1.** (**A**) S2→S1 neurons are labeled with tdTomato following rAAV2-retro-Syn-Cre injection into wS1 of an Ai9 reporter mouse. (**B**) Example confocal image showing tdTomato-labeled neurons in wS2. (**C**) Number of retrogradely labeled S2→S1 neurons in wS2, plotted as a function of normalized depth for two individual mice (left and right panels, n = 1,266 and 1,056 neurons, respectively). Prominent retrograde labeling is evident in L4.

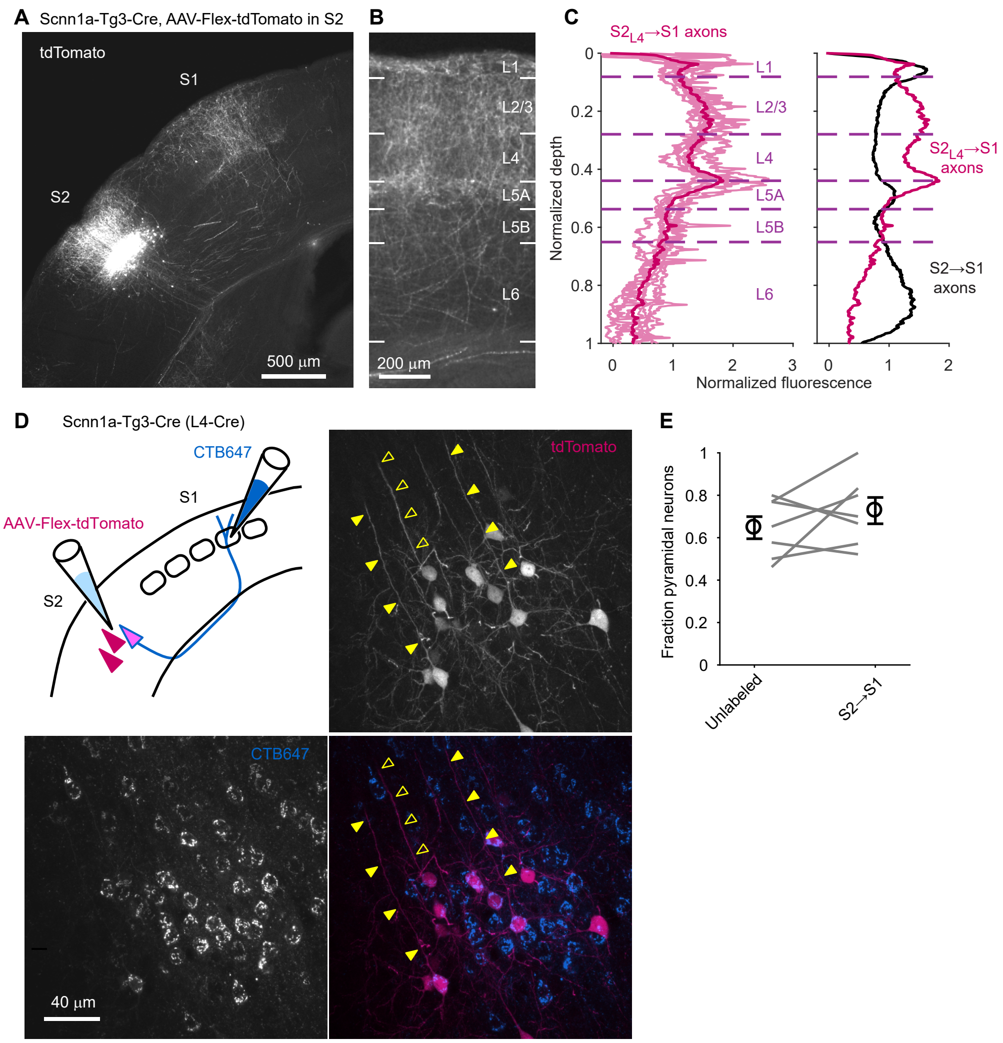


**Figure S4. $S2_{L4}$→S1 neurons largely project to superficial S1 and have pyramidal morphology, related to Figure 1.** (**A**) Example coronal section showing tdTomato fluorescence in an Scnn1a-Tg3-Cre mouse injected with a Cre-dependent tdTomato virus (AAV-Flex-tdTomato) into wS2. Fluorescence from wS2 L4 neurons expressing tdTomato is evident in superficial wS1. (**B**) Zoomed image of wS1. (**C**) *Left*, Normalized $S2_{L4}$→S1 axonal fluorescence measured in wS1, shown for individual mice (light magenta curves, n = 8) and the mean (dark magenta). *Right*, Mean $S2_{L4}$→S1 data (magenta) replotted together with the mean from Figure1D (black), to allow comparison of the $S2_{L4}$→S1 and total S2→S1 projection patterns. (**D**) *Top left*, Schematic of experiment to examine the morphology of $S2_{L4}$→S1 neurons. S2→S1 neurons were retrogradely labeled with CTB-Alexa647, and $S2_{L4}$ neurons were sparsely labeled using injections of diluted Cre-dependent tdTomato AAV virus (AAV-Flex-tdTomato) in Scnn1a-Tg3-Cre mice. *Top right,* Example confocal image from wS2 showing putative apical dendrites of neurons co-labeled with CTB and tdTomato (closed arrowheads) and a tdTomato-labeled neuron not labeled by CTB (open arrowheads). *Bottom left,* Same field of view but showing CTB-Alexa647 signal only. *Bottom right*, Overlay of tdTomato and CTB-Alexa647 images. (E**)** Fraction of tdTomato-positive neurons in wS2 that had clear pyramidal morphology for individual mice (gray lines, n = 7) and the means (± SEM; 0.65 ± 0.05% and 0.73 ± 0.06% for unlabeled and CTB-labeled neurons, respectively; p = 0.29, paired t-test)..

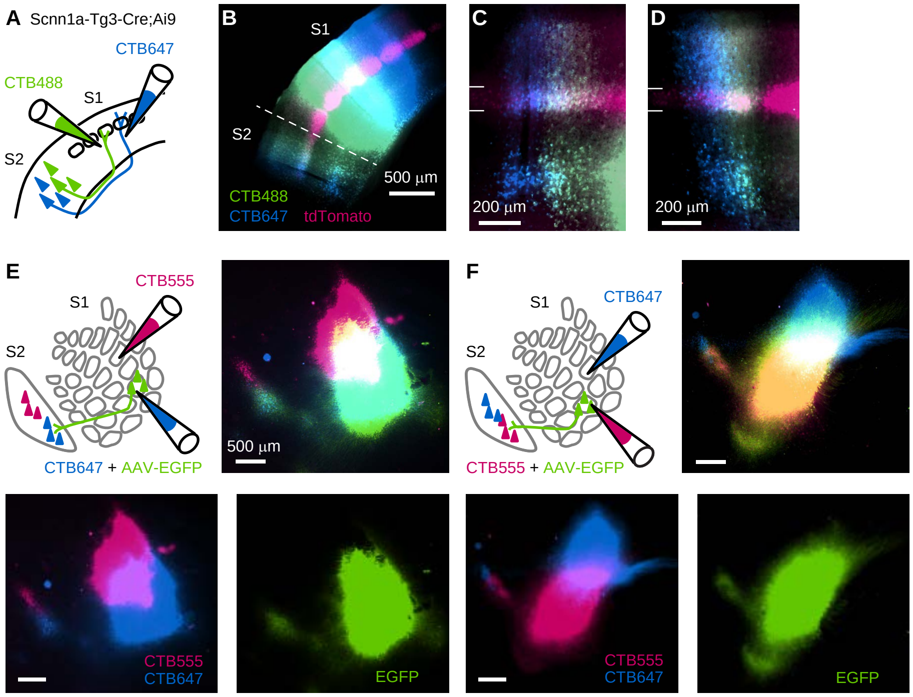


**Figure S5. Additional examples of dual CTB-Alexa injection results, related to Figure 2. (A)** Schematic of experiment. Two colors of CTB-Alexa were injected in different barrel columns of wS1 in a mouse (Scnn1a-Tg3-Cre;Ai9) expressing tdTomato in L4 neurons, to retrogradely label neurons in wS2. (**B**) Coronal section from experiment depicted in (**A**), showing CTB-Alexa488 and CTB-Alexa647 fluorescence at neighboring injection sites in wS1, and locations of retrograde labeling in wS2. (**C**) Zoom of section from (**B**), showing retrogradely labeled site in wS2. Neighboring patterns of CTB-Alexa488 and CTB-Alexa647 are evident, including among L4 neurons. L4 was determined by tdTomato fluorescence and is indicated by horizontal white lines. (**D**) Same as (**C**) but for a different mouse. (**E**) Same as Figure 2D but for a different mouse. (**F**) Same as Figure 2D but for a different mouse.

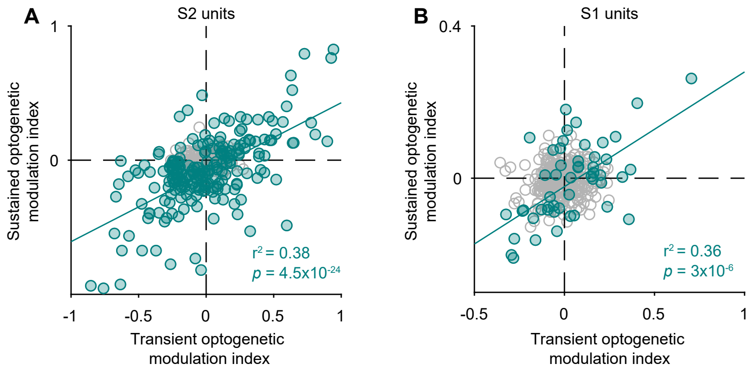


**Figure S6. Optogenetic modulation indices from transient and sustained portions of stimulus are correlated, related to Figure 5.** (**A**) Optogenetic modulation index calculated from transient and sustained phases of the stimulus for S2 units. The transient phase corresponds to the first 100 ms of LED illumination, from -200 ms to -100 ms relative to whisker stimulus onset. The sustained phase corresponds to an identical period as the 500 ms whisker stimulus. Units with optogenetic modulation index significantly different from 0 in either the transient or sustained phases are plotted in light green; other S2 units are plotted in gray. Linear regression line (light green) was fitted to the significant units (n = 212 significant units out of 393 total, 14 recordings from 6 mice). (**B**) Same as (**A**) but for units from S1 (n = 50 significant units out of 347 total, 16 recordings from 6 mice).

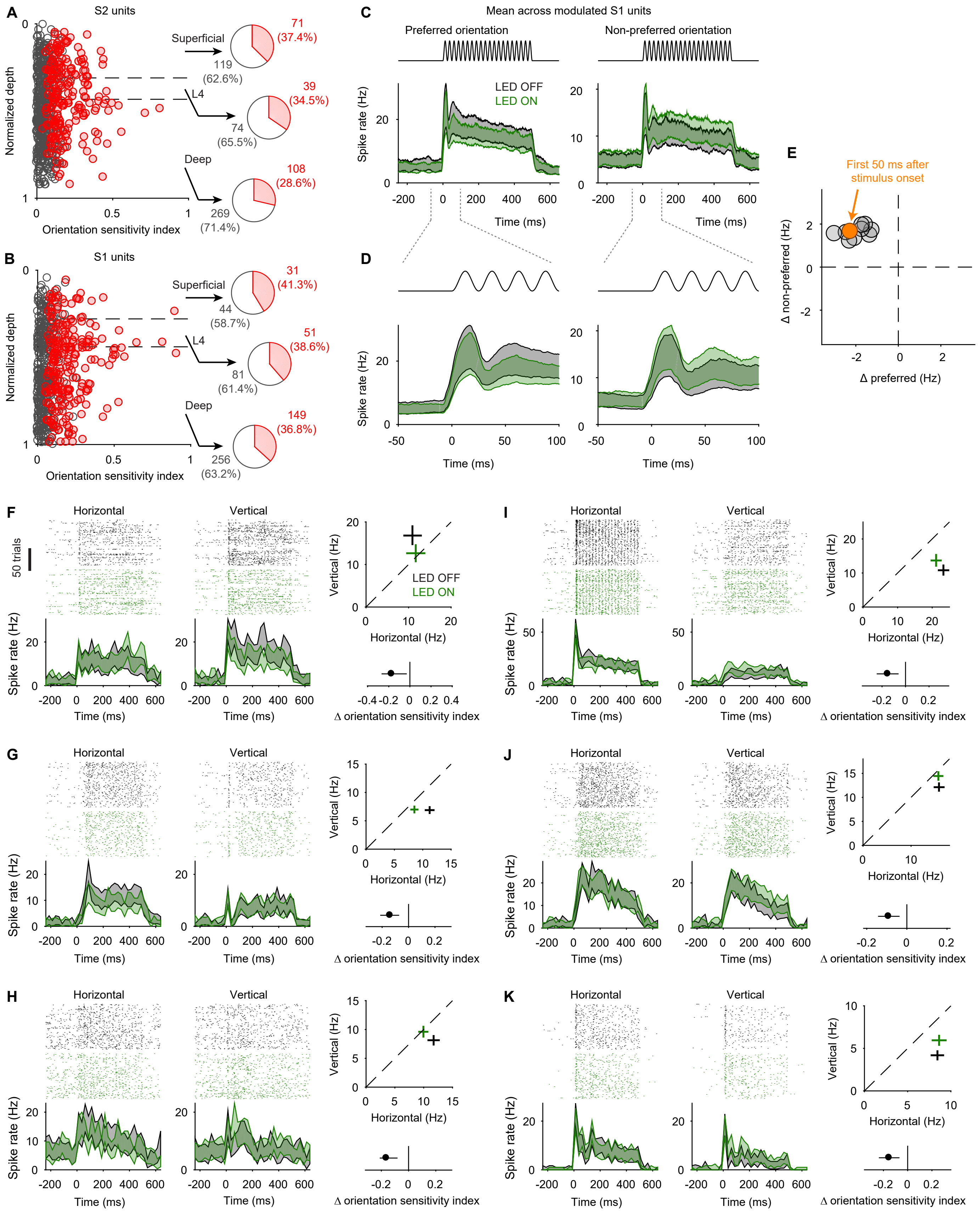


**Figure S7. *OSI* for all S2 and S1 units, time course of effect of $S2_{L4}$ inhibition on S1, and additional examples of S1 units with orientation sensitivity decreased by $S2_{L4}$ inhibition, related to Figure 7.** (**A**) *OSI* for each whisker-responsive S2 unit plotted as a function of normalized depth within cortex. Units with significant orientation sensitivity are plotted in red. Boundaries of cortical layer 4 estimated by current source density analysis are indicated with horizontal dashed lines. (Continued)

Pie charts show percentages of units with significant orientation sensitivity for L4 and layers above ("superficial") and below ("deep") L4. Units are pooled across ChR2 and eArch3.0 experiments (n = 612 units, 27 recordings from 12 mice). (**B**) Same as (**A**) but for whisker-responsive S1 units (n = 680 units, 30 recordings from 13 mice). (**C**) Mean PSTHs for wS1 units with a significant decrease in *OSI* during $S2_{L4}$ inhibition (n = 34; same units as depicted with blue shading in Figure 7J). Separate PSTHs are shown for responses to whisker deflections along the preferred (left) and non-preferred (right) orientations, and with (green) and without (black) $S2_{L4}$ inhibition. (**D**) Same PSTHs as in (**C**), but zoomed to show the first 100 ms after whisker stimulus onset. Effects of $S2_{L4}$ inhibition are evident in this early window. (**E**) Change in mean spike rate during optogenetic inhibition of $S2_{L4}$ for preferred and non-preferred whisker deflection orientations, shown as the mean across wS1 units (n = 34; same as in (**C**)) in consecutive 50 ms time bins beginning at stimulus onset. The first time bin (0 to 50 ms after stimulus onset) is indicated in orange. The effect of $S2_{L4}$ inhibition occurs in all time bins. (**F**) Example S1 unit showing reduction in response to preferred (vertical) orientation during $S2_{L4}$ inhibition. (**G**) Example S1 unit showing reduction in response to preferred (horizontal) orientation during $S2_{L4}$ inhibition. (**H**) Example S1 unit showing reduction in response to preferred (horizontal) orientation during $S2_{L4}$ inhibition. (**I**) Example S1 unit showing increase in response to non-preferred (vertical) orientation during $S2_{L4}$ inhibition. (**J**) Example S1 unit showing increase in response to non-preferred (vertical) orientation during $S2_{L4}$ inhibition. (**K**) Example S1 unit showing increase in response to non-preferred (vertical) orientation during $S2_{L4}$ inhibition. (**F**-**K**) Conventions as in Figure 7F,G.
